# Coordinated regulation of the metaboproteome by Hsp90 chaperones controls metabolic plasticity

**DOI:** 10.1016/j.isci.2026.116105

**Published:** 2026-09-10

**Authors:** R. Felipe Perez, Gianna L. Mochi, Julia K. Burkacki, Victoria Zoccoli-Rodriguez, Landon D. Arcadi, Sarah J. Backe, Joel R. Wilmore, Mark R. Woodford

**Affiliations:** 1 Department of Urology, SUNY Upstate Medical University, Syracuse, NY 13210, USA; 2 Department of Biochemistry & Molecular Biology, SUNY Upstate Medical University, Syracuse, NY 13210, USA; 3 Upstate Cancer Center, SUNY Upstate Medical University, Syracuse, NY 13210, USA; 4 Department of Microbiology and Immunology, SUNY Upstate Medical University, Syracuse, NY 13210, USA

**Keywords:** TRAP1, Hsp90, molecular chaperone, mitochondria, metabolism, metaboproteome

## Abstract

Heat shock protein 90 (Hsp90) chaperones participate in the stabilization and activation of hundreds of proteins, thereby acting as signaling hubs. A mitochondrial subpopulation of Hsp90 has been previously described; however, little is known about its role in metabolism. Here, we showed that loss of individual Hsp90 isoforms differentially affects oxygen consumption and metabolic flexibility. Proteomic and metabolomic evaluation demonstrated that Hsp90 regulates the mitochondrial metabolic network, including respiration, fatty acid oxidation, and redox homeostasis. Loss of the mitochondrial chaperone tumor necrosis factor receptor-associated protein 1 (TRAP1) induced compensatory binding of Hsp90s to TRAP1-dependent proteins, indicating a mechanism for the role of Hsp90 chaperones in metabolic reprogramming. When considered with previous findings, a temporal pattern of regulation emerges whereby Hsp90s control the transcription, translation, import, and assembly of mitochondrial protein complexes. Our findings expand the scope of Hsp90-regulated processes and potentially inform the effects of isoform-specific Hsp90 inhibitors on metabolic reprogramming in cancer and other diseases.

## Highlights

• TRAP1, Hsp90α, and Hsp90β differentially impact cell metabolism
• Proteins that comprise the “metaboproteome” are Hsp90 interactors
• Hsp90 isoform loss disrupts redox homeostasis
• Chaperone compensation reprograms metabolism

## Graphical abstract

**Figure:**
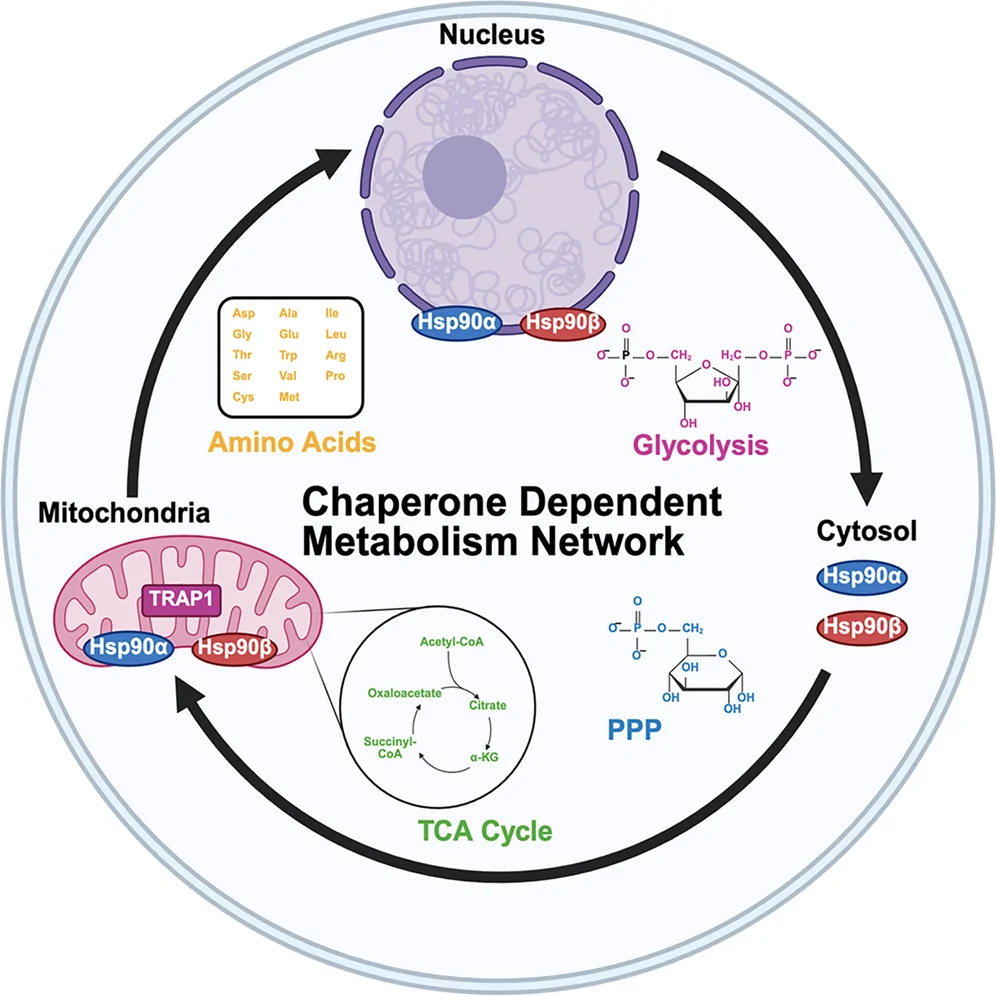

## Teaser

Biological sciences; Molecular biology; Metabolomics

## Introduction

Metabolism is governed by the activity, abundance, and localization of enzymes whose functions are intimately linked to their structural integrity. Metabolic homeostasis depends not only on gene expression and allosteric regulation but also on the correct folding, maturation, and turnover of the metabolic proteome.^1^^,^^2^ Recent studies have highlighted the responsiveness of metabolism to proteostatic stress, including the activation of adaptive pathways such as the unfolded protein response (UPR^3^^,^^4^), the integrated stress response (ISR^5^^,^^6^), and autophagy.^7^ However, the extent to which molecular chaperones control metabolic protein function remains undetermined.

Heat shock protein 90 (Hsp90) is a family of molecular chaperones that use the energy of ATP hydrolysis to regulate the stabilization and activation of their substrate proteins, or “clients.”^8^^,^^9^ Hsp90 chaperones include the constitutively expressed Hsp90β, the stress-inducible Hsp90α, and the organelle-restricted isoforms glucose-regulated protein 94 (Grp94; endoplasmic reticulum^10^) and tumor necrosis factor (TNF) receptor-associated protein 1 (TRAP1; mitochondria).^11^ These chaperones undergo a “chaperone cycle,”^8^^,^^9^ which describes the dynamic regulatory interactions that lead to the stabilization and activation of hundreds of client proteins, centering these proteins as hubs of cellular signaling.^12^ By regulating client function, Hsp90s have been shown to directly control many cellular processes, including the cell cycle,^13^^,^^14^ autophagy,^15^ apoptosis,^16^ and stress responsiveness.^17^

Hsp90α and Hsp90β are found abundantly in the cytosol and have been observed to traffic mitochondrial precursor proteins to the translocase of the outer mitochondrial membrane (TOM) complexes for mitochondrial import. The mitochondrial Hsp90, TRAP1, contains an N-terminal mitochondrial targeting signal and has a role in regulating the function of complexes II and III of the electron transport chain (ETC)^18^; however, the broader impact of TRAP1 activity is poorly understood.^19^^,^^20^ Previous works in mammalian cells have observed an intramitochondrial population of Hsp90^18^^,^^21^ that impacts respiration.^22^ Additionally, a role has been attributed to Hsp90 in regulating the mitochondrial permeability transition,^23^^,^^24^ a precipitating event in certain cell death pathways.^25^ Despite this, the interplay between Hsp90α, Hsp90β, and TRAP1 in the regulation of mitochondrial metabolism has not been investigated.

In this study, we set out to systematically investigate the impact of individual Hsp90 chaperone knockouts on the cellular metabolic network in human cells. We employed both pharmacological and genetic strategies to observe the effects of chaperone function loss on the metabolome and “metaboproteome,” or the protein network that supports metabolism in the cytosol and mitochondria. By mapping metabolic flux changes in response to specific chaperone perturbations, we find that loss of Hsp90α or Hsp90β reprograms the mitochondrial proteome, resulting in dysregulation of the metabolite landscape spanning both intra- and extra-mitochondrial metabolic pathways.

## Results

### Hsp90s control mitochondrial structure and function

**Figure 1:**
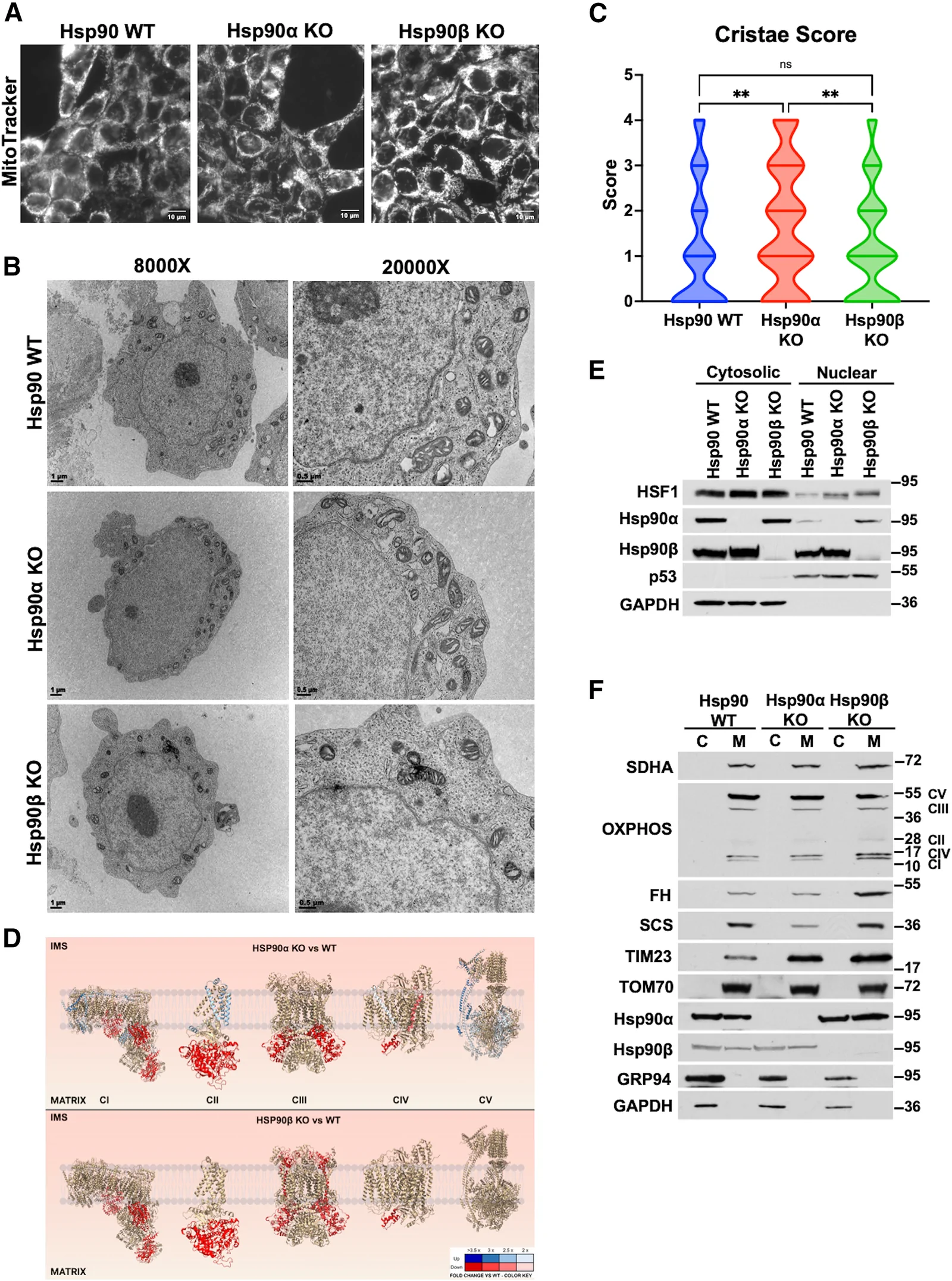
Hsp90s control mitochondrial structure and function (A) WT, Hsp90α KO, and Hsp90β KO HEK293 cells were incubated with MitoTracker Green (50 nM, 20 m). Scale bars, 10 μm. (B) Representative transmission electron microscopy images of WT, Hsp90α KO, and Hsp90β KO HEK293 cells. Scale bars, 1 μm (8000 ×) or 0.5 μm (20000 ×). (C) Mitochondria cristae score calculated based on images collected in Figure 1B. ∗∗ *p* < 0.005 using Welch’s ANOVA. (D) Schematic representation of electron transport chain subunits whose mRNA levels are impacted by loss of Hsp90α or Hsp90β. Data are presented as fold change KO/WT. Red subunits indicate decreased mRNA expression, while blue subunits represent increased mRNA expression. PDB: CI, 5xtd; CII, 8gs8; CIII, 5xte; CIV, 5xth; and CV, 8h9s. (E) Cytosolic and nuclear fractions of WT, Hsp90α KO, and Hsp90β KO HEK293 cells were immunoblotted with the indicated antibodies. p53 represents a control nuclear protein. (F) Cytosolic (C) and mitochondrial (M) fractions collected from WT, Hsp90α KO, and Hsp90β KO HEK293 cells were immunoblotted with the indicated antibodies. GAPDH represents a cytosolic control protein, and GRP94 represents a control for ER contamination.

To understand the function of Hsp90 in the mitochondria, we first examined the impact of specific Hsp90α and Hsp90β knockout (KO) on mitochondrial network organization. In the presence of MitoTracker, we observed that while mitochondria from wild-type (WT) HEK293 cells were small and round, mitochondria from Hsp90α and Hsp90β KO cells were more filamentous and tubular (Figures 1A, S1A, and S1B). Further, tetramethylrhodamine, methyl ester (TMRM) uptake indicated that mitochondrial membrane potential was decreased in Hsp90 KO cells (Figure S1C). We therefore asked whether the loss of membrane potential could be explained by changes in mitochondrial ultrastructure. Interestingly, we found that mitochondria from Hsp90α KO cells were morphologically distinct from those in WT and Hsp90β KO cells, exhibiting increased mitochondrial number and cristae score (a measure of cristae abundance, definition, and organization^26^) (Figures 1B, 1C, and S1D–S1F). Interestingly, mitochondria from Hsp90β KO cells were more similar to WT counterparts, both in gross morphology and cristae score (Figures 1B, 1C, and S1D–S1F). Collectively, these data demonstrate that loss of Hsp90 impacts mitochondrial morphology.

The ETC provides cristae structure.^27^ Given the observed variability in cristae organization in the cell models, we asked whether ETC assembly was impacted following Hsp90 KO. To visualize intact ETC complexes, we performed blue native PAGE. We observed a pronounced reduction in ETC complex formation in Hsp90α KO mitochondria and a modest reduction in Hsp90β KO mitochondria (Figure S1G). We next asked whether these differences could be the result of a failure of Hsp90 KO cells to transcribe nuclear-encoded mitochondrial proteins (Figure 1D). Using a respiration-targeted PCR microarray, we found that mRNA levels of many soluble ETC subunits were reduced following loss of Hsp90α or Hsp90β, while many membrane-bound subunits exhibited elevated transcript levels only in Hsp90α KO (Figures 1D; Table S1). Based on the inconsistent effects on respiration-associated transcript levels, we tested whether Hsp90 loss potentially impacted transcription factor translocation to the nucleus. We first evaluated heat shock factor-1 (HSF1), as HSF1 activity has been shown to effect transcriptional responses downstream of stress caused by loss of Hsp90 function^28^^,^^29^ and is implicated in regulating mitochondrial chaperone expression.^30^ We observed HSF1 induction in both Hsp90 KO cell lines, but not the control transcription factor p53 (Figure 1E), demonstrating that transcription factor activation was not a generalized phenomenon. Therefore, we sought to identify which transcription factors could be responsible for regulating ETC transcription. The ChIP-X Enrichment Analysis v.3 (ChEA3; https://maayanlab.cloud/chea3/^31^) transcription factor prediction tool identified several proteins potentially involved in regulating the expression of these ETC subunits (Table S2). Interestingly, we did not identify HSF1 among the top predicted hits from our dataset. However, some of the top hits associated with expression of the downregulated mitochondrial proteins are Hsp90 clients, including c-Myc,^32^ ERα,^33^ ATF3,^34^ and REST,^35^ suggesting one potential Hsp90-dependent mechanism for transcriptional tuning of respiration.

Despite these transcriptional changes, we observed a modest elevation in mitochondrial expression and import of ETC subunits (Figures 1F, S1H, and S1I) and a pronounced induction of the mitochondrial membrane transporter TIM23 (Figure 1F), indicative a compensatory proteostatic response. Interestingly, we observed inconsistent expression of the tricarboxylic acid (TCA) cycle enzymes fumarase (FH) and succinyl-CoA synthetase (SCS) (Figure 1F), hinting that Hsp90 KO cells may experience chronic metabolic dysregulation resulting from loss of Hsp90. Taken together, these data suggest that despite the lack of individual Hsp90 isoforms and the observed perturbations in transcription, the minimal protein chaperoning and trafficking requirements to support mitochondrial function were met.

### Loss of Hsp90α or Hsp90β differentially impacts mitochondrial metabolism

**Figure 2:**
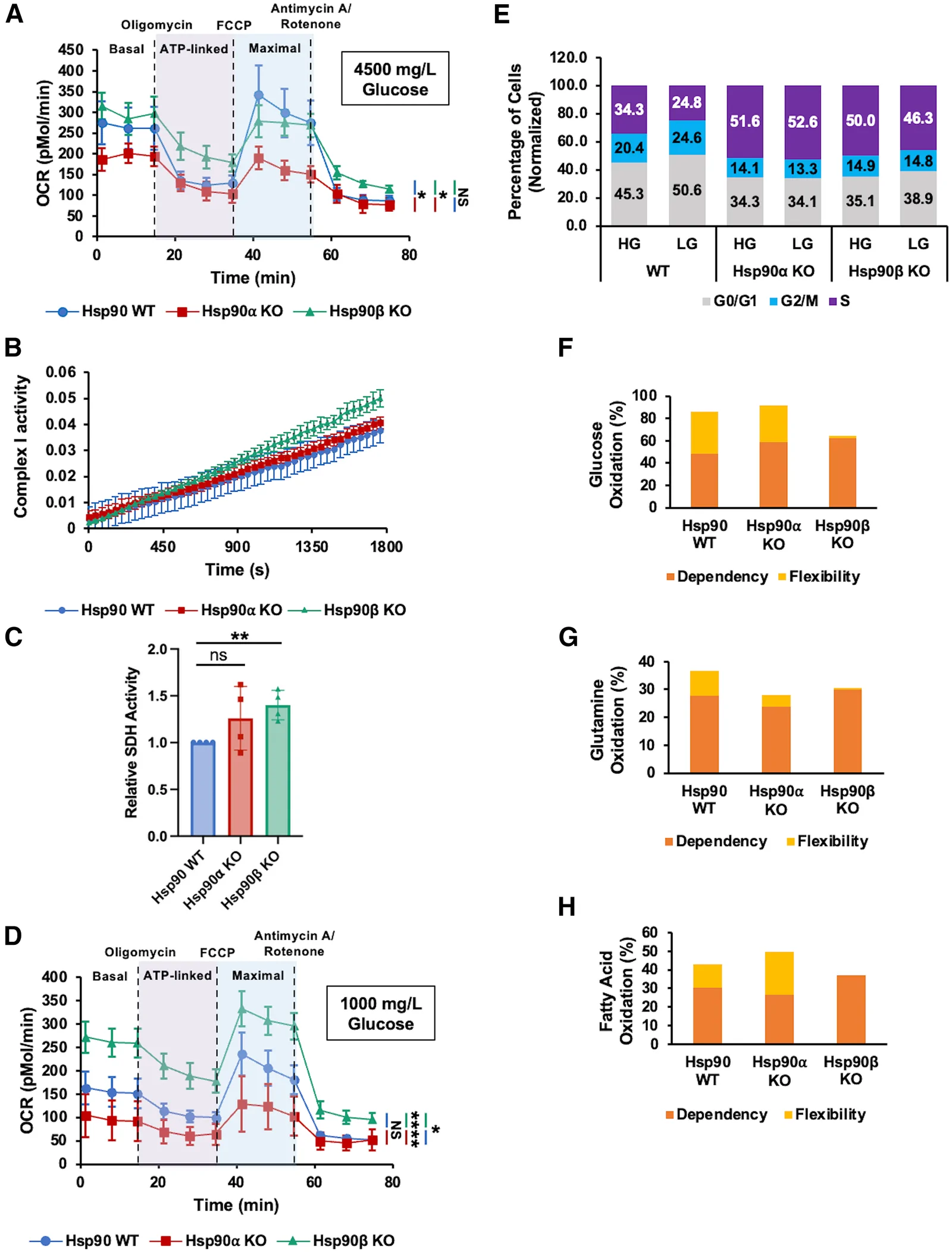
Loss of Hsp90α or Hsp90β differentially impacts mitochondrial metabolism (A) Oxygen consumption rate (OCR) measured by mitochondrial stress test of WT, Hsp90α KO, and Hsp90β KO HEK293 cells grown in DMEM containing 4,500 mg/L glucose. Data are presented as mean ± s.d.; ∗ *p* < 0.05 for WT vs. Hsp90α KO and Hsp90α KO vs. Hsp90β, using 2-way ANOVA with Tukey’s post-test. (B) 25 μg of intact mitochondria isolated from WT, Hsp90α KO, and Hsp90β KO HEK293 cells were assayed for complex I activity *in vitro* using validated commercial kits. Data are presented as mean ± s.d. (C) 25 μg of intact mitochondria isolated from WT, Hsp90α KO, and Hsp90β KO HEK293 cells were assayed for complex II activity *in vitro* using validated commercial kits. Data are presented as mean ± s.d.; ∗∗*p* < 0.005, determined using Student’s *t*-test. (D) OCR measured by mitochondrial stress test of WT, Hsp90α KO, and Hsp90β KO HEK293 cells grown in DMEM containing 1,000 mg/L glucose. Data are presented as mean ± s.d.; ∗*p* < 0.05 for WT vs. Hsp90β KO, ∗∗∗∗*p* < 0.0001 for Hsp90α KO vs. Hsp90β KO, determined using 2-way ANOVA with Tukey’s post-test. (E) BrdU incorporation assay of WT, Hsp90α KO, and Hsp90β KO HEK293 cells grown in DMEM containing 4,500 mg/L (HG) or 1,000 mg/L (LG) glucose for 24 h and incubated with 10 μM BrdU for an additional 2 h. (F–H) Fuel flex assay of WT, Hsp90α KO, and Hsp90β KO HEK293 cells grown in DMEM containing 4,500 mg/L glucose. Glucose (F), glutamine (G), or fatty acid (H) oxidation dependency or flexibility was calculated according to the manufacturer protocol and is presented as stacked bar charts. Top-yellow: flexibility. Bottom-orange: dependency.

The differences in ETC assembly and TCA cycle enzyme expression prompted us to hypothesize that Hsp90 KO cell lines exhibited distinct respiratory output. We first evaluated this by culturing cells in high-glucose medium (4,500 mg/L), which reduces dependence on glucose oxidation. In this condition, Hsp90α KO cells exhibited a significantly reduced oxygen consumption rate profile (OCR, a surrogate for mitochondrial respiration), while WT and Hsp90β KO cells demonstrated similar basal and maximal OCR (Figure 2A). Oligomycin treatment reduced WT OCR (but not that of Hsp90β KO) to the levels of Hsp90α KO cells, indicating that this difference was largely due to compromised ATP-linked respiration (Figure 2A). To understand the origin of dysregulated ATP-linked respiration, we measured the activity of ETC complex I and II *in vitro*. We observed that WT and Hsp90α KO mitochondria had similar levels of ETC complex I and II activity, while activity in Hsp90β KO cells was significantly increased (Figures 2B and 2C). We then measured OCR after culturing the cells in low-glucose medium (1,000 mg/L), which increases dependence on respiration. Compared to WT, Hsp90α KO cells demonstrated reduced respiration, while Hsp90β KO cell OCR was dramatically elevated (Figures 2D and S2A). Collectively, we observed that Hsp90β KO cells maintained an elevated respiratory phenotype in both conditions, while WT and Hsp90α KO cell respiration was significantly elevated in the high-glucose condition. Importantly, this metabolic phenotype is independent of cell proliferation, as both Hsp90α and Hsp90β KO lines accumulated in S-phase at the expense of G0/G1 and G2/M populations and showed decreased overall proliferation relative to WT counterparts (Figures 2E and S2B). These data indicate that while Hsp90 isoforms have distinct impacts on respiration, Hsp90β facilitates metabolic flexibility.

To explore the observed differences in metabolic flux caused by altered glucose abundance, we performed a fuel flex assay, which quantifies the relative dependence and flexibility of cells for particular metabolic fuels. Our data clearly demonstrated that WT and Hsp90α KO cells exhibited high metabolic flexibility and a similar distribution between oxidation of glucose, glutamine, or fatty acids (Figures 2F–2H and S2C–S2H). Hsp90β KO cells demonstrated a marked preference for glucose oxidation with little inherent flexibility (Figures 2F–2H and S2C–S2H), suggesting that while the presence of Hsp90α supports respiration, Hsp90β may regulate metabolic adaptability.

### Hsp90 isoforms regulate diverse aspects of the cellular metabolic network

**Figure 3:**
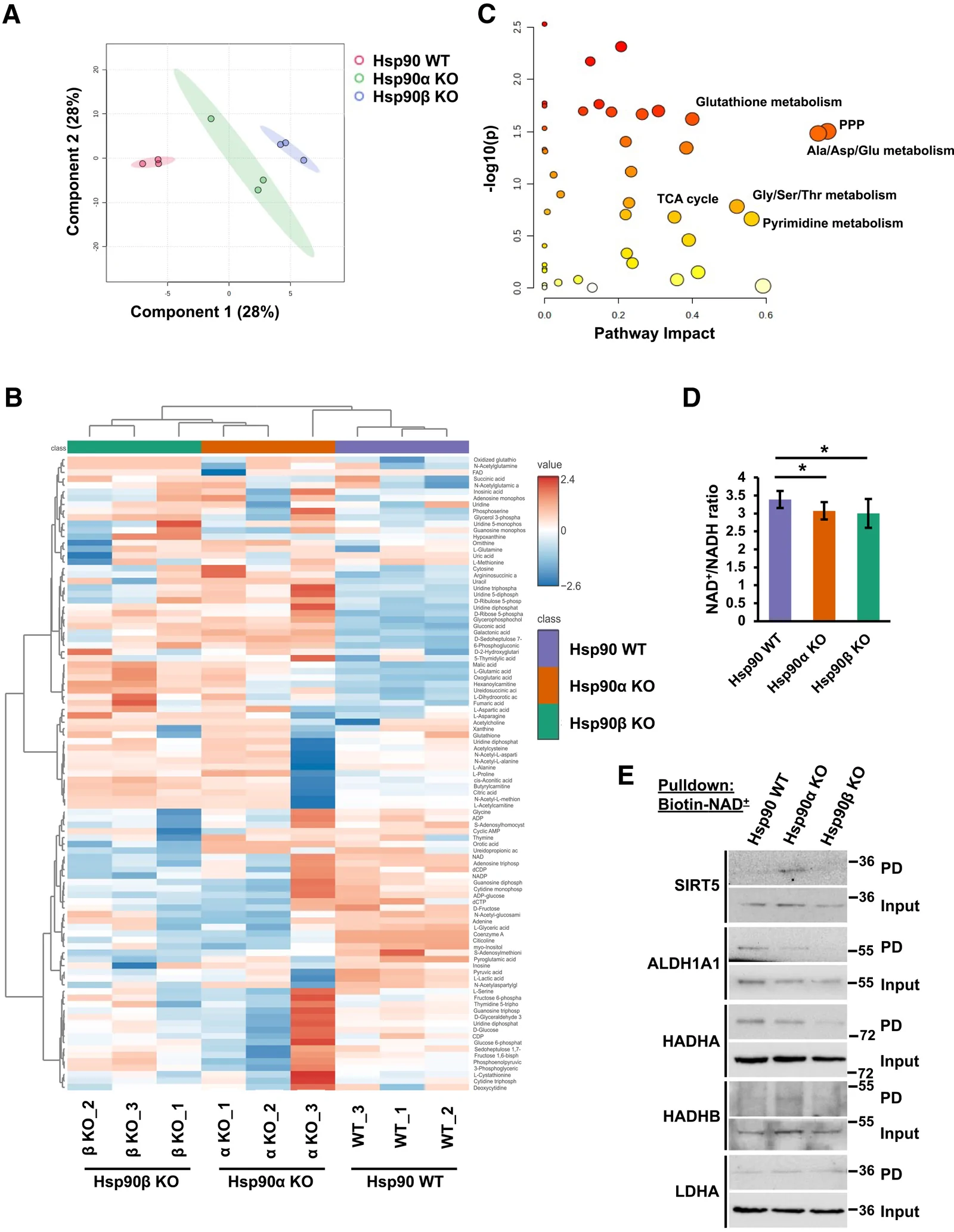
Hsp90 isoforms regulate diverse aspects of the cellular metabolic network (A) Principal component analysis of WT, Hsp90α KO, and Hsp90β KO HEK293 showing the relationship between targeted metabolite composition across biological replicates. (B) Heatmap dendrogram of targeted metabolite abundance across each replicate of WT, Hsp90α KO, and Hsp90β KO HEK293 metabolite extract. (C) Pathway impact analysis of targeted metabolites isolated from WT, Hsp90α KO, and Hsp90β KO HEK293 cells. Figures 3A–3C are derived from the same experimental dataset. *n* = 3 biological replicates per condition. (D) NAD^+^/NADH ratio was calculated based on NAD^+^/NADH-Glo Assay performed in WT, Hsp90α KO, and Hsp90β KO HEK293 cells. Data are presented as mean ± s.d.; ∗ *p* < 0.05 as determined by Student’s *t*-test. (E) Streptavidin pulldown of biotin-NAD^+^ from WT, Hsp90α KO, and Hsp90β KO HEK293 cell lysate was immunoblotted with the indicated antibodies.

To begin to understand the mechanisms underlying metabolic reprogramming following Hsp90α or Hsp90β loss, we performed a high-throughput metabolomic analysis on whole-cell metabolite extracts (Figures 3A, 3B, and S3A). Our data showed that the most significantly dysregulated pathways between WT and Hsp90 KO cells were the pentose phosphate pathway (PPP) and Ala/Asp/Glu metabolism (Figures 3C and S3B–S3E). Hsp90 inhibition has been previously shown to decrease nucleotide biosynthesis in diffuse large B-cell lymphoma cells by reducing the levels of fructose-6-phosphate, ribose-5-phosphate, and glyceraldehyde-3-phosphate, among others.^36^ Interestingly, constitutive Hsp90 KO did not consistently impact the abundance of these metabolites in our dataset. On the contrary, Hsp90 KO largely corresponded to increased abundance of PPP intermediates (Figure S3D). Significantly, most of the identified metabolites relating to Ala/Asp/Glu metabolism were elevated in both Hsp90 KO cell lines, with the exception of pyruvate, which was decreased in both KO cell lines (Figures S3E and S3F). Despite this, we observed increased levels of several TCA cycle metabolites in Hsp90 KO cells, with Hsp90β KO cells generally exhibiting a greater increase than Hsp90α KO (Figure S3F). These data indicate that the presence of Hsp90α meaningfully promotes glucose oxidation.

Further, we observed a marked reduction in NAD^+^ levels in both Hsp90 KO cell lines, particularly in Hsp90β KO cells (Figure S4A). In agreement, NAD^+^/NADH ratio was significantly decreased in Hsp90 KO cells (Figure 3D). To understand the impact of this dysregulation on cells, we examined the levels and NAD^+^-binding ability of a subset of NAD^+^-binding proteins. We found that the mitochondrial proteins hydroxyacyl-CoA dehydrogenase, α-subunit (HADHA) and aldehyde dehydrogenase 1A1 (ALDH1A1) exhibited decreased levels and biotin-NAD^+^ binding, while HADHB and sirtuin-5 (SIRT5) exhibited elevated expression and biotin-NAD^+^ binding specifically in Hsp90α KO cells (Figures 3E and S4B). Importantly, the cytosolic NAD^+^-binding protein LDHA was unaffected (Figures 3E and S4B), suggesting a mitochondria-specific effect on NAD^+^-binding protein expression and function. Taken together, these observations suggest an altered redox environment following loss of Hsp90, potentially contributing to the loss of metabolic flexibility in Hsp90β KO cells.

### The mitochondrial metaboproteome is controlled by Hsp90

**Figure 4:**
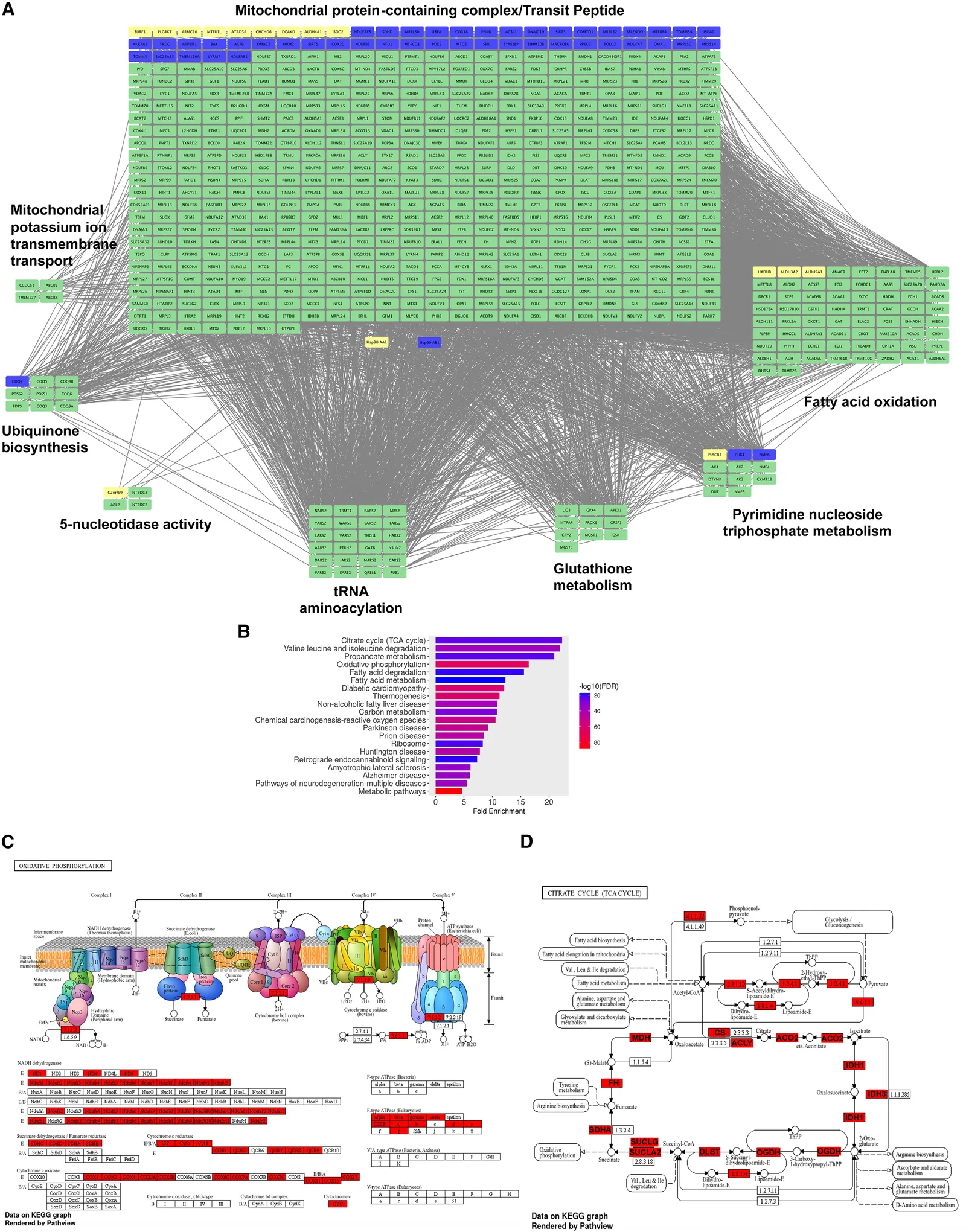
The mitochondrial metaboproteome is controlled by Hsp90 (A) Protein interaction network of mitochondrial Hsp90α (yellow) and Hsp90β (blue). Proteins found in both interactomes are green. A complete list of interacting proteins can be found in Table S3. (B) Kyoto Encyclopedia of Genes and Genomes (KEGG) enrichment analysis of mitochondrial Hsp90-interacting proteins. (C) Hsp90 interactors mapped onto a schematic of the ETC. (D) Hsp90 interactors mapped onto a schematic of the TCA cycle pathway. Red-labeled proteins in (C and D) were identified the interactome in Figure 4A. Figures 4A–4D are derived from the same experimental dataset. *n* = 2 biological replicates per condition.

To further understand the impact of Hsp90 loss on mitochondrial metabolism, we asked whether any mitochondrial proteins were clients of mitochondrial Hsp90s. To accomplish this, we isolated Hsp90-binding proteins by immunoprecipitating Hsp90α or Hsp90β from the mitochondria of WT HEK293 cells. We observed mitochondrial Hsp90α and Hsp90β interacting with more than 500 intramitochondrial proteins, with some modest preference for individual Hsp90 isoforms (Figures 4A and S5A; Table S3). Interestingly, Hsp90β alone interacted with several subunits of the ETC complexes, including NDUFA7, NDUFB1, NDUFAB1, and NDUFAF5 (CI); SDHD (CII); LYRM7 (CIII); COX18, COX20, and MT-CO1 (CIV); and ATP5IF1 (CV), as well as COQ7, involved in ubiquinone biosynthesis (Figure 4A). Overall, Hsp90 interactors were significantly enriched for proteins involved in respiration, as well as amino acid and fatty acid metabolism (Figures 4B and S5B). Significantly, the majority of ETC subunits were identified in the Hsp90 interactome, including those of the F-type, but not V-type ATPase (Figure 4C), as well as at least one subunit of each TCA cycle complex (Figure 4D). In concert with our prior observations, these data hint at one potential mechanism by which Hsp90β regulates mitochondrial respiration.

The NAD^+^-dependent deacylases SIRT3/5 were also identified as Hsp90β-specific interactors, drawing a correlation with the increased NAD^+^-binding capacity of SIRT5 in Hsp90α KO cells and significantly decreased NAD^+^ levels in Hsp90β KO cells. Similarly, Hsp90α specifically interacted with HADHB and two ALDH family members, integral players in fatty acid oxidation (FAO). In support of this, Hsp90α KO cell OCR responded to the FAO inhibitor etomoxir (ET), in contrast to WT and Hsp90β KO cells, indicative of a potentially important role for Hsp90α in regulating FAO (Figure S5C).

### Overlapping Hsp90 interactions promote mitoproteostasis

**Figure 5:**
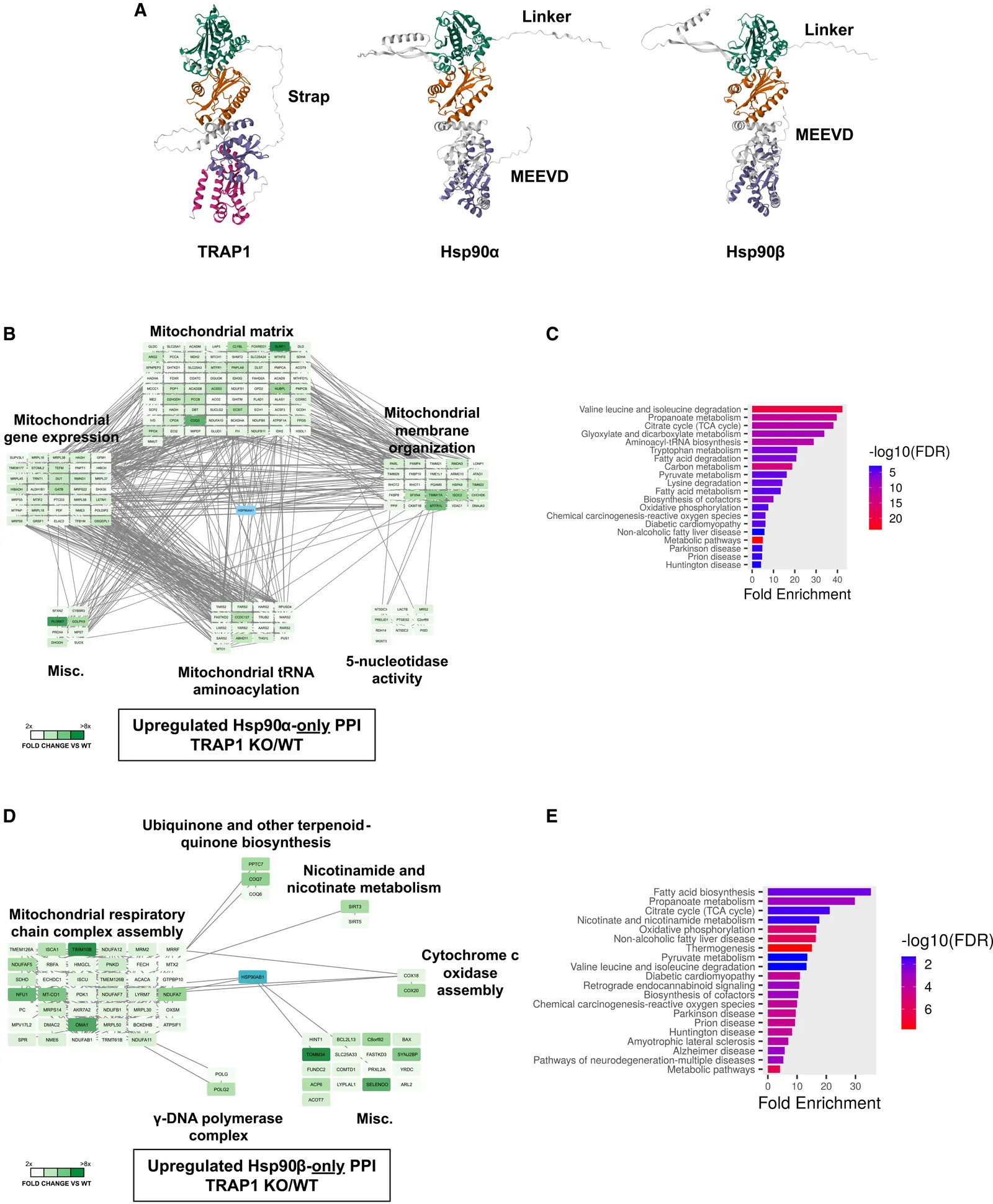
Chaperone compensation supports mitoproteostasis (A) Alphafold^39^^,^^40^ structure prediction of TRAP1, Hsp90α, and Hsp90β. Colored portions represent discrete structural domains (N-domain: green, Hsp90 large and small middle domains: orange and gray, TRAP1 large and small middle domains: orange and purple, Hsp90 C-domain: purple, and TRAP1 C-domain: pink). (B) Interaction network of proteins whose interaction with Hsp90α, but not Hsp90β, is upregulated in TRAP1 KO HEK293 cells. Increased green color intensity corresponds to degree of interaction upregulation. (C) Kyoto Encyclopedia of Genes and Genomes (KEGG) enrichment analysis of proteins whose interaction with Hsp90α, but not Hsp90β, is upregulated in TRAP1 KO HEK293 cells. (D) Interaction network of proteins whose interaction with Hsp90β, but not Hsp90α, is upregulated in TRAP1 KO HEK293 cells. Increased color intensity corresponds to degree of interaction upregulation. (E) KEGG enrichment analysis of proteins whose interaction with Hsp90β, but not Hsp90α, is upregulated in TRAP1 KO HEK293 cells. Figures 5B–5E are derived from the same experimental dataset. *n* = 3 biological replicates per condition.

The mitochondria-dedicated Hsp90 isoform TRAP1 has an established role in controlling mitochondrial respiration by regulating the activity and assembly of the ETC.^19^^,^^20^ TRAP1 exhibits distinct structural features from Hsp90α and Hsp90β, suggestive of significant functional divergence, including a marked reduction in the length of the charged N-C linker and a loss of the C-terminal MEEVD sequence, a binding motif for interaction with tetratricopeptide repeat-containing proteins^37^^,^^38^ (Figure 5A). Therefore, we asked whether the three Hsp90 chaperones exhibited unique or overlapping interactors that might explain their respective functions in regulating respiration. To accomplish this, we isolated Hsp90α or Hsp90β from the mitochondria of HEK293 cells and compared their interactors in WT and TRAP1 KO cells. We initially sought to understand the degree to which TRAP1 loss induced compensatory binding of Hsp90 to mitochondrial proteins. To quantify this, we devised a chaperone compensation score, which allowed us to compare the proportion of upregulated protein interactions between conditions, expressed as a percentage of total interactions. A high compensation score (> 50%) indicates an increased likelihood of functional redundancy between chaperones, while a low compensation score likely indicates poor chaperone functional overlap. We observed that Hsp90α and Hsp90β both compensate for TRAP1 loss by demonstrating a > 2-fold increase in binding to the majority of detected proteins (Figures S6A–S6D; Tables S4, S5, S6, and S7), with Hsp90α displaying a greater breadth of compensation (78% of interactions increased vs. 59%, based on > 700 interactions), perhaps due to its intrinsic stress-responsive nature (Figures 5B and S6E). Individually, Hsp90α took on increased interaction with proteins involved in amino acid metabolism and aminoacyl-tRNA biosynthesis (Figures 5B and 5C; Table S8) and acquired *de novo* interactions with proteins regulating nicotinate and nicotinamide metabolism, oxidative phosphorylation, and mitoribosome function (Figures S6F and S6G; Table S9). Compensatory Hsp90β interaction demonstrated a preference for proteins involved in fatty acid biosynthesis and nicotinate and nicotinamide metabolism, while both Hsp90 isoforms demonstrated enrichment for TCA cycle proteins in the absence of TRAP1 (Figures 5D and 5E; Table S10). Importantly, this compensatory mitochondrial Hsp90 chaperone activity was correlated with increased cell proliferation, as TRAP1 KO cells demonstrated a marked increase in S phase cells compared to WT controls (Figure S6H).

To understand whether Hsp90 chaperone compensation was bidirectional, we next compared TRAP1 interactors in WT, Hsp90α KO, and Hsp90β KO cells. Our data showed that in the absence of Hsp90α or Hsp90β, TRAP1 took on additional interactors previously observed in the Hsp90α or Hsp90β interactomes, suggesting a degree of functional redundancy that was slightly elevated in Hsp90α KO cells (71% of interactions increased vs. 60%, based on ∼50 interactors; Figure S7A). Interestingly, proteins associated with fatty acid and amino acid metabolism were significantly enriched for TRAP1 protein-protein interactions (PPIs) in Hsp90 KO cells (Figures S7B–S7D), while TCA cycle proteins were significantly underrepresented (Figures S7E and S7F; Tables S11, S12, S13, S14, S15, S16, and S17). We examined a subset of these interactors by immunoblot and observed that differences in PPIs were not due to changes in the abundance or mitochondrial import of chaperone-interacting proteins (Figures S8A and S8B). Overall, we identified an order of magnitude more interactors for Hsp90s compared to TRAP1, resulting in a 14-fold increase in upregulated Hsp90 PPIs in TRAP1 KO compared to TRAP1 PPIs in Hsp90 KO cells (Figures S8C and S8D). Taken together, these data suggest that Hsp90α and Hsp90β may have roles distinct from each other but overlapping with TRAP1, and further that Hsp90s are significantly more capable of compensating for TRAP1 than the inverse.

### Chaperone compensation supports metabolic flux

**Figure 6:**
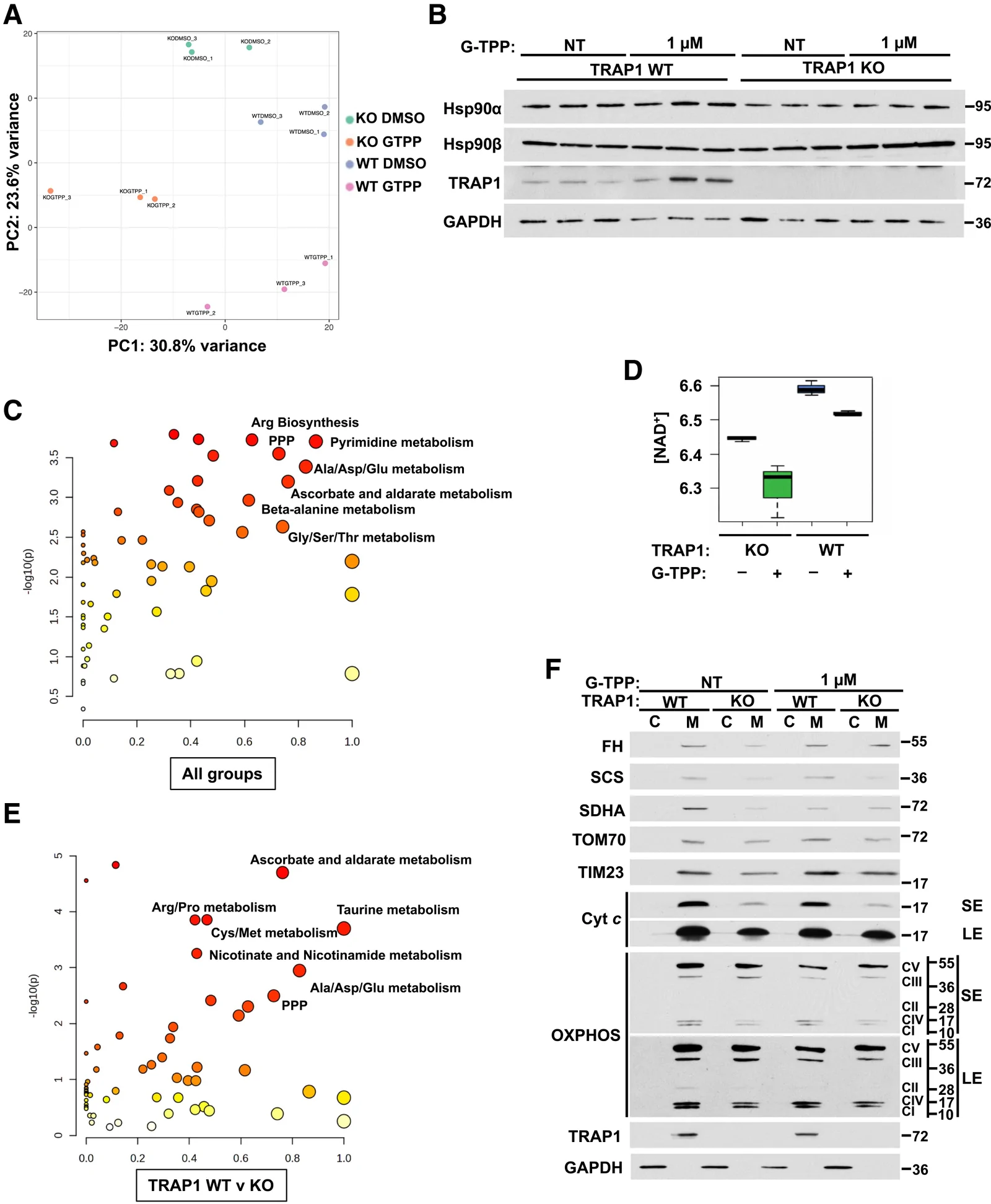
Chaperone compensation supports metabolic flux (A) Principal component analysis of WT or TRAP1 KO HEK293 cell metabolite composition across biological replicates in the presence or absence of the mitochondrial Hsp90 inhibitor 1 μM G-TPP for 2 h. (B) Immunoblot of protein fractions of WT or TRAP1 KO HEK293 samples submitted for metabolomic analysis in Figure 6A. (C) Pathway impact analysis of metabolites isolated from WT or TRAP1 KO HEK293 cells in the presence or absence of 1 μM G-TPP for 2 h. (D) NAD^+^ abundance in metabolite extracts from WT or TRAP1 KO HEK293 cells in the presence or absence of 1 μM G-TPP for 2 h. Data are presented as log₁₀-transformed concentrations. (E) Direct comparison of pathway impact of metabolites isolated from WT or TRAP1 KO HEK293 cells. Figures 6A–6E are derived from the same experimental dataset. *n* = 3 biological replicates per condition. (F) Immunoblot of metabolic enzymes from cytosolic (C) or mitochondrial (M) fractions of WT or TRAP1 KO HEK293 cells in the presence or absence of 1 μM G-TPP for 2 h. SE, short exposure; LE, long exposure.

We next hypothesized that Hsp90 isoform compensation would manifest in differential regulation of mitochondrial respiration under stress. To test this, we treated WT and TRAP1 KO cells with a short pulse of a sub-lethal dose of the mitochondria-targeted Hsp90 inhibitor gamitrinib-TPP (G-TPP^41^). Surprisingly, principal component analysis (PCA) of the metabolomics data showed that DMSO-treated WT and TRAP1 KO cells (green and blue) clustered more closely to each other than to their G-TPP-treated counterparts (orange and pink), suggesting the presence of a meaningfully interconnected Hsp90 chaperone network disrupted by G-TPP treatment (Figures 6A, 6B, and S9A).

We explored TRAP1- and G-TPP-dependent differences in metabolic profile using Metaboanalyst 6.0.^42^ Among all four groups, PPP function and amino acid metabolism were among the primary pathways exhibiting significant alterations (Figure 6C). Furthermore, TRAP1 loss reduced cellular NAD^+^ levels, which was exacerbated by mitochondrial Hsp90 inhibitor G-TPP treatment (Figure 6D). These observations are consistent with our metabolomic data from Hsp90 KO cells (Figure 3), indicating that altered metabolic flux and redox balance are in part dependent on mitochondrial Hsp90 function.

Within the untreated groups, we found that TRAP1 loss was primarily associated with dysregulation of Ala/Asp/Glu, Arg/Pro, and Cys/Met metabolism, as well as ascorbate and taurine metabolism (Figure 6E). Interestingly, we observed that TRAP1 loss appreciably decreased UDP-GlcNAc levels (Figure S9B), consistent with our prior observations demonstrating a relationship between TRAP1 and *O*-GlcNAcylation.^43^ Pyrimidine metabolism was similarly dysregulated in both WT and TRAP1 KO cell lines following G-TPP treatment (Figures S9C, S9D, and S10A–S10F), indicating that this pathway is Hsp90-dependent, but TRAP1 independent. Interestingly, we found that low dose G-TPP treatment had distinct effects on mitochondrial metabolic protein levels in TRAP1 WT and KO cells (Figure 6F). These effects are particularly evident for SDHA and FH, where TRAP1 loss decreases protein levels and G-TPP treatment of these cells provides a partial rescue (Figure 6F). These data indicate that differential chaperone compensation dynamics or chaperoning requirements for these proteins may layer significant nuance onto metabolic flux regulation. Taken together, our data strongly support distinct mechanisms by which Hsp90α, Hsp90β, and TRAP1 control metabolic flux distribution.

## Discussion

Hsp90 chaperones play a significant role in regulating biological processes through their ability to control protein folding, maturation, and activation. To fully understand how Hsp90 chaperone function impacts metabolism, we applied integrated high-resolution proteomic, metabolomic, and transcript-level datasets along with functional assays. We have demonstrated both specific and overlapping roles for three Hsp90 chaperones in controlling metabolite flux and metabolic flexibility. Perhaps most importantly, we have quantitatively identified a set of chaperone-protein interactions and metabolite signatures in response to loss of individual Hsp90 chaperones or inhibition of the mitochondrial Hsp90 chaperone pool.

Our data clearly demonstrate a role for intra- and extra-mitochondrial Hsp90 function in regulating nucleotide, amino acid, and fatty acid metabolism. In agreement with our work, previous proteomic studies have found Hsp90 interaction with enzymes in glycolysis, the TCA cycle, and one carbon metabolism,^44^^,^^45^ and a pronounced effect of Hsp90 inhibitors on both glycolysis and respiration in cancer cells.^46^^,^^47^ However, relative to cytosolic Hsp90 clients, chaperone dependency among mitochondrial proteins is poorly understood. We have described mitochondrial Hsp90 interactors and categorized their interactions with individual Hsp90 chaperones. Based on our observation of altered chaperone compensation capacity, it is conceivable that Hsp90 and TRAP1 may have different roles with respecting to “holding” or “folding” proteins in the mitochondria, and potentially even function in a sequential manner. Unraveling this interplay will be key to understanding the application and efficacy of isoform-specific Hsp90 inhibitors.^48^^,^^49^^,^^50^^,^^51^

The findings presented here implicate Hsp90s as critical regulators of NADH homeostasis through several interrelated mechanisms. Hsp90 or TRAP1 loss disrupts NAD^+^/NADH ratio by reducing NAD^+^ levels and compromising the ability of mitochondrial NAD^+^-binding proteins to interact with NAD^+^. Interestingly, our data showed three distinct patterns for NAD^+^-binding proteins in response to Hsp90 KO: (1) decreased expression/NAD^+^-binding of HADHA and ALDH1A1, (2) increased expression/NAD^+^-binding of SIRT5 and HADHB in Hsp90α KO only, and (3) no change for the cytosolic protein LDHA. While it is tempting to speculate on whether direct Hsp90 binding impacts NAD^+^-binding capacity, there was no clear pattern in our data. HADHB was identified in our proteomics dataset as an Hsp90α-specific interactor, while SIRT5 was identified as an Hsp90β-specific interactor. HADHA interacts with both Hsp90 isoforms, while ALDH1A1 was not identified in our interactome. Collectively, these data suggest that in addition to NAD^+^ abundance, the ability of Hsp90 to impact NAD-binding may depend on a number of factors, including whether the protein is an Hsp90 client. NADH levels are also controlled by the malate-aspartate shuttle (MAS), which is responsible for transporting reducing equivalents into the mitochondria for oxidative phosphorylation.^52^ Our data show that GOT2, the mitochondrial isoform of glutamic-oxaloacetic transaminase and linchpin of the MAS, is among Hsp90 and TRAP1 interactors. Furthermore, malate and aspartate levels are upregulated while 2-oxoglutarate levels are decreased, both in Hsp90 and TRAP1 KO cells as well as in response to G-TPP treatment, indicating MAS dysregulation. Interestingly, minimal changes in NADH levels were observed, but NADP^+^ abundance mirrored NAD^+^. These key observations suggest that Hsp90s regulate MAS function and potentially also contribute to disrupted redox homeostasis across the cell.

Despite limited organelle-specific resolution of metabolite abundance and Hsp90 function, some of our results are informative. For example, we found that mitochondrial Hsp90 inhibitor G-TPP treatment significantly reduced orotate formation. The mitochondrial enzyme DHODH catalyzes orotate production and is a significantly increased interactor of Hsp90α in TRAP1 KO cells. Notably, this is the only step of pyrimidine biosynthesis that occurs in the mitochondria. This pathway is significantly impacted in both Hsp90 and TRAP1 KO cells, as well as in response to G-TPP treatment, in agreement with previous observation.^36^ However, the relative contributions of each chaperone to the folding, maturation, and activation of enzymes in this pathway remain unresolved. Several similarly compelling vignettes can be made for other metabolite-enzyme pairs based on our data, highlighting the potentially significant metabolic dependencies of Hsp90 chaperone activity.

Our data showed significant compensatory mitochondrial protein binding following individual Hsp90 isoform loss. This observation suggests three potential scenarios regarding Hsp90 chaperone function: (1) Hsp90 chaperones participate in an ordered client maturation process. This concept is supported by a relatively larger pool of interacting proteins identified for Hsp90s compared to TRAP1, which is supported by previous work.^53^ This observation may indicate that Hsp90 could have less specificity for binding to mitochondrial proteins than TRAP1 and may interact with these proteins “earlier” in their maturation process. (2) TRAP1 and Hsp90 have significant functional redundancy and collaborate to mature mitochondrial proteins. While the PPIs of these three Hsp90 isoforms cannot be directly compared using the data presented here, we observed significant overlap between identified interactors of Hsp90 and TRAP1 across the two proteomics datasets. (3) In many cell types, TRAP1 represents the major mitochondrial Hsp90 chaperone, with other Hsp90s recruited in cancer and potentially other stress conditions.^23^ Therefore, it remains possible that in HEK293 cells, as well as cells undergoing significant mitochondrial or proteostatic stress, the mitochondrial Hsp90 network is expanded to effect a survival response. Understanding this functional overlap in basal conditions is an essential next step to understanding mitochondrial Hsp90 chaperone dynamics.

Post-translational modification (PTM) is a major regulatory mechanism for Hsp90 chaperones,^54^^,^^55^ and can also potentially contribute to localization or retention in specific subcellular compartments. Previous work has shown that nitration of Hsp90-Y33 promotes mitochondrial translocation of Hsp90, with a concomitant negative effect on respiration.^22^ Tyrosine nitration is induced by oxidative stress, suggesting a potential feedback mechanism regulating Hsp90 control of oxidative metabolism. A mechanistically similar PTM, nitrosylation, has been observed to regulate TRAP1 activity and stability, with an opposing effect on respiration.^56^^,^^57^ The ability to simultaneously modulate intramitochondrial Hsp90 isoform function under oxidative conditions may provide cells the ability to fine-tune metabolism under stress. Whether additional PTMs are involved in Hsp90 mitochondrial trafficking or activity is unknown.

Dysregulation of chaperone function has been implicated in various diseases, including cancer,^58^ neurodegenerative disorders,^59^^,^^60^ and cystic fibrosis,^61^ where proper chaperone function is critical for preserving protein homeostasis in cells. As such, Hsp90 inhibitors have consistently been observed to have strong anti-proliferative and anti-cancer effects.^62^ Our data indicate a substantial impact of Hsp90 loss on nucleotide, amino acid, and fatty acid biosynthesis, suggesting that the decreased proliferation and accumulation of Hsp90 KO cells in S-phase that we observed may result from a lack of essential macromolecules required for cell division. This study was inspired by previous work from Kang et al.^23^ demonstrating the increased presence of mitochondrial Hsp90 in cancer cells. While HEK293 cells are not cancerous, studies have observed phenotypically similar metabolic and growth behaviors, including an overwhelming preference for LDHA-directed conversion of pyruvate to lactate,^63^ elevated PPP flux,^64^^,^^65^ and anchorage-independent growth.^66^ Therefore, the present study is likely to yield results consistent with cancer models that exhibit similar metabolic and growth characteristics. This work will inform future studies that explore the role of Hsp90 in the regulation of mitochondrial function in cancer cells. Moreover, understanding the individual roles of these chaperones in the regulation of disease-associated pathways will help to refine therapies based on targeting specific chaperone isoforms.

### Limitations of the study

The current study has four noteworthy limitations that are essential to acknowledge when interpreting the data presented here. First, the use of constitutive Hsp90 isoform KO cells allows for the possibility of cellular adaptation that may not faithfully phenocopy the effects of acute Hsp90 inhibition or attenuation. Secondly, proteomics data presented in this study have been performed in the absence of protease treatment, which would remove extramitochondrial proteins as well as those associated with the outer mitochondrial membrane. Therefore, some observed Hsp90 interactions are likely to be the product of Hsp90 associated with mitochondrial protein import complexes. Thirdly, the work presented here was conducted exclusively in HEK293 cells. While these cells are widely used to model chaperone function, other cultured cell lines, primary cell cultures, or 3D culture models have not been evaluated in this study and may exhibit variability with respect to mitochondrial Hsp90 residence and function. Finally, isoform-specific small molecule Hsp90 inhibitors with mitochondrial tropism are not currently available, preventing direct evaluation of cells containing a single functional Hsp90 isoform within the mitochondria.

## Resource availability

### Lead contact

Further information and requests for resources and reagents should be directed to and will be fulfilled by the lead contact, Mark R Woodford (woodform@upstate.edu).

### Materials availability

This study did not generate new unique reagents.

### Data and code availability

• Data reported in this study are available in the supplemental information and can also be shared by the lead contact upon request.
• This article does not report original code.
• Mass spectrometry proteomics data are publicly available at the ProteomeXchange Consortium via the PRIDE^67^ partner repository with the dataset identifier PXD069999.
• Metabolomics datasets are publicly available at the Metabolomics Workbench with the dataset identifier PR002775 and can be accessed directly via its project DOI: https://doi.org/10.21228/M8ZK26.

## Acknowledgments

The authors thank Mehdi Mollapour, Dimitra Bourboulia, and Gennady Bratslavsky for support, mentorship, and stimulating scientific discussions. The authors acknowledge Benjamin Zink, manager of the 10.13039/100010956Upstate Medical University Transmission Electron Microscopy Core for TEM imaging services, and Xiaowen Wang for technical assistance. The authors also acknowledge Guoan Zhang, director of the 10.13039/100020424Weill Cornell Medicine Proteomics and Metabolomics Core for mass spectrometry analysis and invaluable technical input. This work was supported by funds from 10.13039/100010956SUNY Upstate Medical University, 10.13039/100010956Upstate Urology, The Upstate Cancer Center, and 10.13039/100017776The Upstate Foundation.

## Author contributions

Conceptualization, M.R.W.; methodology, R.F.P., G.L.M., V.Z.-R., L.D.A., S.J.B., and M.R.W.; investigation, R.F.P., G.L.M., J.K.B., V.Z.-R., L.D.A., S.J.B., and M.R.W.; writing – original draft, M.R.W.; writing – review and editing, R.F.P., G.L.M., S.J.B., J.R.W., and M.R.W.; funding acquisition, M.R.W.; resources, M.R.W.; supervision, J.R.W. and M.R.W.

## Declaration of interests

The authors compare no competing interests.

## STAR★Methods

### Key resources table

**Table:** 

REAGENT or RESOURCE	SOURCE	IDENTIFIER
**Antibodies**		

AGK	Cell Signaling	RRID: AB_3739972
ALDH1A1	Cell Signaling	RRID: AB_2797805
ATP5A1	Cell Signaling	RRID: AB_2925225
Cyt *c*	Cell Signaling	RRID: AB_2637071
DLAT	Cell Signaling	RRID: AB_2797893
FH	Cell Signaling	RRID: AB_11178522
GAPDH	Cell Signaling	RRID: AB_2756824
GOT2	Cell Signaling	RRID: AB_3662816
GRP94	Cell Signaling	RRID: AB_823506
HADHA	abcam	RRID: AB_2263836
HSF1	Cell Signaling	RRID: AB_2798072
Hsp90α	Cell Signaling	RRID: AB_11217436
Hsp90β	Cell Signaling	RRID: AB_10548761
LDHA	Cell Signaling	RRID: AB_2066887
OXPHOS	abcam	RRID: AB_2629281
p53	Cell Signaling	RRID: AB_331743
SCS	Cell Signaling	RRID: AB_10889930
SDHA	Cell Signaling	RRID: AB_2750900
SIRT5	Cell Signaling	RRID: AB_2716763
TIM23	Cell Signaling	RRID: AB_3739973
TOM70	Cell Signaling	RRID: AB_3411916
TRAP1	Cell Signaling	RRID: AB_2800183

**Chemicals, peptides, and recombinant proteins**		

MitoTracker Green	Invitrogen	M7514
TMRM	Invitrogen	T668
Gamitrinib-TPP	MedChemExpress	HY-102007A
BrdU	BD Biosciences	552598
WST Reagent	abcam	ab155902
^13^C_6_ glucose	Millipore-Sigma	660663
Biotin-NAD^+^	R&D Systems	6573

**Critical commercial assays**		

Complex I activity assay	abcam	ab109721
Complex II activity assay	abcam	ab228560
Seahorse cell mito stress test assay	Agilent	103015-100
Seahorse Fuel Flex assay	Agilent	103260-100
Seahorse fatty acid oxidation assay	Agilent	103672-100
NAD^+^/NADH-Glo assay	Promega	G9071
RT^2^ Profiler PCR Array Human Mitochondrial Energy Metabolism Pathway Plus (PAHS-008YD-6)	Qiagen	330231 D-6

**Deposited data**		

Metabolomics dataset	Metabolomics Workbench	PR002775
Proteomics dataset	PRIDE Repository	PXD069999

**Experimental models: Cell lines**		

HEK293T WT	ATCC	CRL-3216
HEK293T Hsp90α KO	Laboratory of Didier Picard	N/A
HEK293T Hsp90β KO	Laboratory of Didier Picard	N/A
HEK293T TRAP1 KO	Laboratory of Len Neckers	N/A

**Software and algorithms**		

ImageJ	Schneider et al.^68^	https://imagej.net/ij/
MetaboAnalyst 6.0	Pang et al.^42^	https://www.metaboanalyst.ca
Cytoscape v3.10.3	Shannon et al.^69^	https://cytoscape.org
STRING v12	Szklarczyk et al.^70^	https://www.string-db.org
ShinyGO v0.80	Ge at al^71^	https://bioinformatics.sdstate.edu/go80/

### Experimental model and study participant details

#### Cell lines

HEK293T Hsp90 KO cells were a generous gift of Didier Picard (University of Lausanne) and were reported in.^72^ HEK293T TRAP1 KO were a graciously provided by Len Neckers (National Cancer Institute) and were reported in.^53^ Cells were cultured in Dulbecco’s Modified Eagle Medium (DMEM; Sigma-Aldrich) supplemented with 10% fetal bovine serum (FBS; Gibco) at 37 °C in a CellQ incubator at 5% CO2. HEK293 cells are female. The Hsp90 KO cell lines have not been authenticated, but we validated as KO by immunoblot and mass spectrometry, and tested negative for mycoplasma.

### Method details

#### Mitochondrial isolation

Cells were placed on ice and washed once with ice-cold PBS (Millipore-Sigma). Following thorough aspiration to remove PBS, 100 μL of fresh Isotonic Buffer (250 mM Sucrose, 10 mM Tris–HCl pH 7.4, cOmplete protease inhibitor cocktail (Millipore-Sigma), and PhosSTOP (Millipore-Sigma)) was added and cells were scraped and collected in a 1.7 mL Eppendorf tube. The cell suspension was then transferred to a cold Dounce homogenizer and disrupted with 10 strokes of a tight-fitting pestle (Ace Glass). Homogenate was transferred back to a 1.7 mL Eppendorf tube and centrifuged at 600 × g for 10 min to pellet cell debris. Supernatant was transferred to a fresh 1.7 mL Eppendorf tube and centrifuged at 10,000 × g for 10 min to pellet mitochondria. The supernatant from this spin is the cytosolic fraction. Mitochondrial pellet was gently re-suspended in 50 μL fresh Isotonic Buffer for downstream applications.

#### Nuclear fractionation

Cells were placed on ice and washed once with ice-cold PBS (Millipore-Sigma). Following thorough aspiration to remove PBS, 100 μL of fresh cytoplasmic extract (CE) buffer (10 mM HEPES, 60 mM KCl, 1 mM EDTA, 0.075% (v/v) NP-40, 1mM DTT and 1 mM PMSF, adjusted to pH 7.6) was added and cells were scraped into a 1.7 mL Eppendorf tube. Cells were incubated on ice for 20 m, with 2 s vortex every 5 m. Nuclei were pelleted by centrifugation at 1500 rpm for 4 m. Cytoplasmic fraction (supernatant) was removed to a clean 1.7 mL Eppendorf tube and nuclei were washed 2*x* with CE buffer without NP-40. Following removal of second wash buffer, nuclei were sonicated in 100 μL nuclear extraction (NE) buffer (20 mM Tris-HCl, 420 mM NaCl, 1.5 mM MgCl_2_, 0.2 mM EDTA, 1 mM PMSF and 25% (v/v) glycerol, adjusted to pH 8.0). Following 10 m centrifugation at 15,000 rpm, soluble nuclear extract was transferred to a clean 1.7 mL Eppendorf tube and fractions were quantified by Bradford Assay (Bio-Rad). Protocol adapted from Rockland Immunochemicals.

#### Protein extraction and immunoblotting

Cultured cells were placed on ice and washed once with ice-cold PBS (Millipore-Sigma). Following thorough aspiration to remove PBS, 200 μL of fresh lysis buffer (20 mM Tris–HCl (pH 7.4), 100 mM NaCl, 1 mM MgCl2, 0.1% NP40, cOmplete protease inhibitor cocktail (Millipore-Sigma), and PhosSTOP (Millipore-Sigma)) was added to the plate and cells were scraped from the plate and collected in a 1.7 mL Eppendorf tube. The cell suspension was sonicated on ice for 2 s using a probe sonicator (QSonica) and centrifuged for 7 min at 15,000 × g to pellet cell debris. The supernatant containing the soluble protein was transferred to a clean 1.7 mL Eppendorf tube and quantified using a Bradford assay (Bio-Rad) according to the manufacturer’s protocol. Nitrocellulose membranes were immunoblotted with primary antibodies followed by the indicated HRP-conjugated secondary antibodies (Cell Signaling Technology).

#### Immunoprecipitations and pulldowns

For endogenous immunoprecipitation, protein lysates or isolated mitochondria were incubated with the indicated antibodies overnight at 4 °C with rotation followed by Protein G agarose (ThermoFisher Scientific) for an additional 1 h at 4 °C with rotation. For biotinylated pulldowns, protein lysates were incubated with indicated concentrations of biotinylated-NAD^+^ (R&D Systems) for 1 h at 4 °C with rotation followed by streptavidin-agarose beads (ThermoFisher Scientific) for 1 additional h at 4 °C with rotation. Agarose pellets were washed 4 times with fresh lysis buffer with vortexing and eluted at 95 °C in 5*x* Laemmli buffer. Precipitated proteins were separated by SDS-PAGE and transferred to nitrocellulose membranes for immunoblotting or submitted for mass spectrometry analysis.

#### Drug treatment

WT and TRAP1 KO HEK293 cells were treated with 1 μM Gamitrinib-TPP (G-TPP; MedchemExpress) for 18 h prior to subcellular fractionation or metabolite extraction.

#### Fluorescence microscopy

WT, Hsp90α KO, and Hsp90β KO HEK293 were incubated with 50 nM of MitoTracker Green (Invitrogen) or tetramethylrhodamine, methyl ester (TMRM; Invitrogen) for 20 m at 37 °C. For the final 10 m, cells were incubated with 10 μg/mL Hoechst 33258. Following incubation, medium was removed and replaced with pre-warmed DMEM + 10% FBS and cells were imaged immediately on an ECHO Revolve R4 Microscopy System (Discover Echo Inc.).

#### Cristae scoring

Mitochondria cristae analysis was conducted using ImageJ^68^ as previously defined in Lam et al.^26^ Mitochondria were quantified and cristae scores were assigned based on cristae abundance and shape. Scores were determined in accordance with published criteria: 0 for no distinct cristae, 1 for >50% of area without cristae, 2 for >25% of area without cristae, 3 for >75% of area with cristae, but irregularly shaped, 4 for many regular cristae in mitochondria area.

#### Transmission electron microscopy

Cells were trypsinized, resuspended in PBS, collected in tubes and centrifuged at 300 *x* g for 10 m to pellet. Cell pellets were washed 2 additional times with PBS for 5 m each with centrifugation (300 *x* g/10 m). Supernatant was removed and replaced with fixative (2.5% Glutaraldehyde + 2.0% Paraformaldehyde in PBS – Electron Microscopy Sciences (EMS), Hatfield PA), and then the cell pellet was resuspended and tubes placed on ice and allowed to fix for 2 h. Cells were then washed 3*x* with PBS for 5 m each with centrifugation (300 *x* g/10 m), enrobed in 4% agarose, and post-fixed for 1.5 h with 1% Osmium Tetroxide in PBS on ice (EMS, Hatfield PA). Cells were then washed 4 times with PBS for 5 m each, placed in a fresh vial containing distilled water, and dehydrated through a graded ethanol series to 100%, after which propylene oxide was exchanged 2 times for 15 m each. Following manufacturers standard protocol, cells were infiltrated with and embedded in Embed-812 epoxy resin (EMS, Hatfield PA). Post polymerization, sample blocks were trimmed to expose the cell pellet, then sectioned at 70 nm with a UC7 ultramicrotome (Leica Microsystems, Wetzler Germany) equipped with a diamond knife (Diatome, Quakertown PA). Sections were collected onto 200 mesh grids, allowed to dry, then counter stained with UranyLess for 4 m and lead citrate for 3 m (EMS, Hatfield PA). Cells were then observed with a JEM-1400 Transmission Electron Microscope (TEM) operated at 80kV (JEOL USA Inc., Peabody MA) and digital images acquired with an Orius SC-1000 CCD camera (Gatan Inc., Pleasanton CA) in the Upstate TEM Core.

#### Blue native page

Mitochondria were isolated as described. 25 μg of mitochondria were prepared by incubation with 1% digitonin in isotonic buffer on ice for 30 m. Samples were then centrifuged at 21000 *x* g for 15 m to pellet insoluble membranes. Native PAGE sample buffer and G-250 were added and samples were loaded onto the gel, then run and stained using the Invitrogen BN-PAGE system, as previously reported.^73^

#### PCR microarray

RNA was isolated from cells using the RNA Isolation kit (Ambion) according to the manufacturer’s protocol. iScript cDNA Synthesis kit was used to generate cDNA according to manufacturer’s protocol. RT^2^ Profiler PCR Array Human Mitochondrial Energy Metabolism Pathway Plus (PAHS-008Y; Qiagen) was performed according to manufacturer’s protocol using 20 ng RNA-equivalent of cDNA. Significant alterations were color-coded according to the following: Positive fold change is colored blue, and downregulation is colored red. Dark blue signifies a fold change exceeding 3.5-fold, while light blue represents a fold change between 2.0 and 2.49-fold. Conversely, gene downregulation is illustrated by the red-colored chains. Similar to upregulation, the shade of red signifies the fold change. Dark red denotes a fold change greater than 3.5, while light red indicates a fold change between 2.0 and 2.49.

#### WST proliferation assay

Cells were plated at 10^5^ cells/well. After 72 h of culture, MTT assay was performed as described using the Quick Cell Proliferation Colorimetric Assay Kit Plus (BioVision).

#### BrdU incorporation assay

Indicated cells were plated at 10^5^/mL in DMEM containing either 4500 mg/L or 1000 mg/L glucose. Following 24 h culture, 10 μM BrdU (BD Biosciences) was added for an additional 2 h. Cells were prepared according to the manufacturer’s protocol and analyzed using a BD Fortessa.

#### Complex II/succinate dehydrogenase activity assay

SDH activity was measured in 25 μg of intact mitochondria using the Colorimetric Succinate Dehydrogenase *in vitro* activity assay (abcam) according to the manufacturer’s protocol and read on a Tecan Spark plate reader.

#### Complex I activity assay

Complex I activity was measured in 25 μg of intact mitochondria using the Colorimetric Complex I *in vitro* activity assay (abcam), according to the manufacturer’s protocol, and read on a Tecan Spark plate reader.

#### Metabolic flux analysis

Cells were seeded at 5 x 10^5^/well in a 96-well assay plate. Following overnight incubation, cells were treated or subject to media changes as indicated for 18 h prior to assay initiation. The indicated assay (Mitochondrial Stress Test, Fuel Flex Assay, Fatty Acid Oxidation Assay) was then carried out on an Agilent Seahorse XFe96 instrument according to the manufacturer’s protocol.

#### Metabolomic analysis

For untargeted metabolomics, cells were cultured in indicated medium for 24 h prior to metabolite extraction. For isotope tracing targeted metabolomics, cells were cultured in low-glucose DMEM supplemented with 3.5 g/L ^13^C_6_ glucose for 30 m prior to metabolite collection. All metabolites were methanol-extracted from cells at 80% confluency. Briefly, cells were washed one time with PBS on wet ice. After thorough aspiration, cells were moved to dry ice and scraped in 700 μL ice-cold 80% methanol. Extract was centrifuged at 14000 *x* g for 20 m. Supernatant was transferred to a new tube and samples were completely dried using a vacufuge. Mass spectrometric analyses were performed at the Weill Cornell Proteomics and Metabolomics Core Facility. The human metabolite database (HMDB) and an in-house metabolite library containing standard retention time information were searched for metabolite identification. Three levels of metabolite identification were reported: 1) identified compounds: definitive identification based on the mass (within 5 ppm) and retention time of authentic chemical standards; 2) putatively annotated compounds by searching HMDB (mass tolerance 5 ppm); 3) compounds with predicted chemical composition based on mass. Relative metabolite quantitation was performed based on peak area for each metabolite. Metabolites present in fewer than 50% of samples were removed prior to analysis. Remaining missing values were imputed using a half-minimum approach. Relative metabolite quantitation was performed based on peak area for each metabolite. Intensity values were evaluated for normality by Shapiro-Wilk test, log transformed and normalized by Pareto scaling, followed by visualization using MetaboAnalyst 6.0 web-based tools. Three biological replicates per condition were analyzed and presented.

#### Proteomic analysis

The indicated proteins were immunoprecipitated from Hsp90 WT, Hsp90α KO, Hsp90β KO, TRAP1 WT and TRAP1 KO HEK293 cells with 2-3 biological replicates per condition. IgG IP was used as a negative control, and proteins identified in negative control samples were removed prior to analysis. The remaining proteins are defined as interactors. Mass spectrometric analyses were performed at the Weill Cornell Proteomics and Metabolomics Core Facility. The identified proteins presented in Figure 4 were cleaned using the following criteria: greater than one unique peptide and present in a minimum of two replicates. The proteins were normalized to the intensities of the precipitated proteins (TRAP1, Hsp90α or Hsp90β) and averaged across replicates. Mitochondrial proteins were validated by cross-referencing to the MitoCarta 3.0 database (Broad Institute), and only those proteins present in MitoCarta were analyzed. Significant fold changes are colored green (greater than 1.9) or red (less than 0.5). Cytoscape v3.10.3^69^ was used to create interactomes based on STRING PPI networks (STRING, version 12.0^70^). ShinyGO v0.80^71^^,^^74^ was used to perform GO/KEGG analyses.

#### Chaperone compensation calculation

Percent chaperone compensation was calculated using the following formula: ((Upregulated PPIs-downregulated PPIs)/Total PPIs)∗100. Chaperone compensation score was calculated by ratiometric comparison as indicated: Upregulated Hsp90 PPI in TRAP1 KO/Upregulated TRAP1 PPI in Hsp90 KO.

#### NAD/NADH-glo assay

Cells were seeded at 10^6^/well in a tissue-culture treated 96-well plate. After 24 h, cells were lysed in 1% DTAB and divided into two groups in a white 96-well plate to measure NAD^+^ and NADH. The assay was then carried out according to the manufacturer’s protocol (Promega) and read on a Tecan Spark plate reader.

### Quantification and statistical analysis

The data presented are the representative of three biological and/or technical replicates, as specified in the Figure Legends. All statistics were performed using GraphPad Prism version 7.00 for Mac (GraphPad Software, La Jolla, California, USA, https://www.graphpad.com). Statistical significance between paired samples was ascertained using parametric Student’s *t*-test. Group comparisons were made using one-way or two-way ANOVA with Tukey’s post-test. Significance was denoted with asterisks in each figure: ∗*p* < 0.05; ∗∗*p* < 0.01; ∗∗∗*p* < 0.001; ∗∗∗∗*p* < 0.0001 or NS (not significant). Error bars represent the standard deviation (s.d.) for three independent experiments, unless otherwise indicated in the Figure Legends.

## Supplemental information

Document S1. Figures S1–S10

Table S1. Fold change abundance of mRNAs (KO/WT) using RT2 Profiler PCR Array Human Mitochondrial Energy Metabolism Pathway Plus

Table S2. Enrichment score prediction of transcription factors targeting significantly impacted genes using ChEA3 - ChIP-X Enrichment Analysis v.3

Table S3. Mitochondrial Hsp90 interactors

Table S4. Upregulated Hsp90α PPI TRAP1 KO/WT

Table S5. Upregulated Hsp90β PPI TRAP1 KO/WT

Table S6. Downregulated Hsp90α PPI TRAP1 KO/WT

Table S7. Downregulated Hsp90β PPI TRAP1 KO/WT

Table S8. Upregulated Hsp90α-only PPI TRAP1 KO/WT

Table S9. Hsp90α PPI acquired in TRAP1 KO

Table S10. Upregulated Hsp90β-only PPI TRAP1 KO/WT

Table S11. Upregulated TRAP1 PPI Hsp90α KO/WT

Table S12. Downregulated TRAP1 PPI Hsp90α KO

Table S13. Upregulated TRAP1 PPI Hsp90α KO only

Table S14. Upregulated TRAP1 PPI Hsp90β KO/WT

Table S15. Upregulated TRAP1 PPI Hsp90β KO only

Table S16. Downregulated TRAP1 PPI Hsp90β KO

Table S17. TRAP1 PPI only in Hsp90 KO

Table S18. Complete metabolomics report related to Figure 3

Table S19. Complete metabolomics report related to Figure 6

## References

[1] Moehle E.A., Shen K., Dillin A. (2019). Mitochondrial proteostasis in the context of cellular and organismal health and aging. J. Biol. Chem..

[2] Baker M.J., Tatsuta T., Langer T. (2011). Quality Control of Mitochondrial Proteostasis. Cold Spring Harb. Perspect. Biol..

[3] Lee J., Ozcan U. (2014). Unfolded Protein Response Signaling and Metabolic Diseases. J. Biol. Chem..

[4] Lin Y.-F., Haynes C.M. (2016). Metabolism and the UPR mt. Mol. Cell.

[5] Labbé K., LeBon L., King B., Vu N., Stoops E.H., Ly N., Lefebvre A.E.Y.T., Seitzer P., Krishnan S., Heo J.-M. (2024). Specific activation of the integrated stress response uncovers regulation of central carbon metabolism and lipid droplet biogenesis. Nat. Commun..

[6] Ryoo H.D. (2024). The integrated stress response in metabolic adaptation. J. Biol. Chem..

[7] Rabinowitz J.D., White E. (2010). Autophagy and Metabolism. Science.

[8] Schopf F.H., Biebl M.M., Buchner J. (2017). The HSP90 chaperone machinery. Nat. Rev. Mol. Cell Biol..

[9] Sahasrabudhe P., Rohrberg J., Biebl M.M., Rutz D.A., Buchner J. (2017). The Plasticity of the Hsp90 Co-chaperone System. Mol. Cell.

[10] Marzec M., Eletto D., Argon Y. (2012). GRP94: An HSP90-like protein specialized for protein folding and quality control in the endoplasmic reticulum. Biochim. Biophys. Acta.

[11] Van Oosten-Hawle P., Bolon D.N.A., LaPointe P. (2017). The diverse roles of Hsp90 and where to find them. Nat. Struct. Mol. Biol..

[12] Taipale M., Jarosz D.F., Lindquist S. (2010). HSP90 at the hub of protein homeostasis: emerging mechanistic insights. Nat. Rev. Mol. Cell Biol..

[13] Mollapour M., Tsutsumi S., Neckers L. (2010). Hsp90 phosphorylation, Wee1 and the cell cycle. Cell Cycle.

[14] Woodford M.R., Backe S.J., Wengert L.A., Dunn D.M., Bourboulia D., Mollapour M. (2021). Hsp90 chaperone code and the tumor suppressor VHL cooperatively regulate the mitotic checkpoint. Cell Stress Chaperones.

[15] Backe S.J., Sager R.A., Heritz J.A., Wengert L.A., Meluni K.A., Aran-Guiu X., Panaretou B., Woodford M.R., Prodromou C., Bourboulia D. (2023). Activation of autophagy depends on Atg1/Ulk1-mediated phosphorylation and inhibition of the Hsp90 chaperone machinery. Cell Rep..

[16] Peng C., Zhao F., Li H., Li L., Yang Y., Liu F. (2022). HSP90 mediates the connection of multiple programmed cell death in diseases. Cell Death Dis..

[17] Zou J., Guo Y., Guettouche T., Smith D.F., Voellmy R. (1998). Repression of Heat Shock Transcription Factor HSF1 Activation by HSP90 (HSP90 Complex) that Forms a Stress-Sensitive Complex with HSF1. Cell.

[18] Altieri D.C., Stein G.S., Lian J.B., Languino L.R. (2012). TRAP-1, the mitochondrial Hsp90. Biochim. Biophys. Acta.

[19] Matassa D.S., Criscuolo D., Avolio R., Agliarulo I., Sarnataro D., Pacelli C., Scrima R., Colamatteo A., Matarese G., Capitanio N. (2022). Regulation of mitochondrial complex III activity and assembly by TRAP1 in cancer cells. Cancer Cell Int..

[20] Sciacovelli M., Guzzo G., Morello V., Frezza C., Zheng L., Nannini N., Calabrese F., Laudiero G., Esposito F., Landriscina M. (2013). The Mitochondrial Chaperone TRAP1 Promotes Neoplastic Growth by Inhibiting Succinate Dehydrogenase. Cell Metab..

[21] Lee C., Park H.-K., Jeong H., Lim J., Lee A.-J., Cheon K.Y., Kim C.-S., Thomas A.P., Bae B., Kim N.D. (2015). Development of a Mitochondria-Targeted Hsp90 Inhibitor Based on the Crystal Structures of Human TRAP1. J. Am. Chem. Soc..

[22] Franco M.C., Ricart K.C., Gonzalez A.S., Dennys C.N., Nelson P.A., Janes M.S., Mehl R.A., Landar A., Estévez A.G. (2015). Nitration of Hsp90 on Tyrosine 33 Regulates Mitochondrial Metabolism. J. Biol. Chem..

[23] Kang B.H., Plescia J., Dohi T., Rosa J., Doxsey S.J., Altieri D.C. (2007). Regulation of Tumor Cell Mitochondrial Homeostasis by an Organelle-Specific Hsp90 Chaperone Network. Cell.

[24] Margineantu D.H., Emerson C.B., Diaz D., Hockenbery D.M. (2007). Hsp90 Inhibition Decreases Mitochondrial Protein Turnover. PLoS One.

[25] Rasola A., Bernardi P. (2007). The mitochondrial permeability transition pore and its involvement in cell death and in disease pathogenesis. Apoptosis..

[26] Lam J., Katti P., Biete M., Mungai M., AshShareef S., Neikirk K., Garza Lopez E., Vue Z., Christensen T.A., Beasley H.K. (2021). A Universal Approach to Analyzing Transmission Electron Microscopy with ImageJ. Cells.

[27] Stroud D.A., Ryan M.T. (2013). Mitochondria: Organization of Respiratory Chain Complexes Becomes Cristae-lized. Curr. Biol..

[28] Pessa J.C., Joutsen J., Sistonen L. (2024). Transcriptional reprogramming at the intersection of the heat shock response and proteostasis. Mol. Cell.

[29] Kijima T., Prince T.L., Tigue M.L., Yim K.H., Schwartz H., Beebe K., Lee S., Budzynski M.A., Williams H., Trepel J.B. (2018). HSP90 inhibitors disrupt a transient HSP90-HSF1 interaction and identify a noncanonical model of HSP90-mediated HSF1 regulation. Sci. Rep..

[30] Katiyar A., Fujimoto M., Tan K., Kurashima A., Srivastava P., Okada M., Takii R., Nakai A. (2020). HSF1 is required for induction of mitochondrial chaperones during the mitochondrial unfolded protein response. FEBS Open Bio.

[31] Keenan A.B., Torre D., Lachmann A., Leong A.K., Wojciechowicz M.L., Utti V., Jagodnik K.M., Kropiwnicki E., Wang Z., Ma’ayan A. (2019). ChEA3: transcription factor enrichment analysis by orthogonal omics integration. Nucleic Acids Res..

[32] Lee J., Zhang L.L., Wu W., Guo H., Li Y., Sukhanova M., Venkataraman G., Huang S., Zhang H., Alikhan M. (2018). Activation of MYC, a bona fide client of HSP90, contributes to intrinsic ibrutinib resistance in mantle cell lymphoma. Blood Adv..

[33] Segnitz B., Gehring U. (1997). The Function of Steroid Hormone Receptors Is Inhibited by the hsp90-specific Compound Geldanamycin. J. Biol. Chem..

[34] Taipale M., Krykbaeva I., Koeva M., Kayatekin C., Westover K.D., Karras G.I., Lindquist S. (2012). Quantitative Analysis of Hsp90-Client Interactions Reveals Principles of Substrate Recognition. Cell.

[35] Orozco-Díaz R., Sánchez-Álvarez A., Hernández-Hernández J.M., Tapia-Ramírez J. (2019). The interaction between RE1-silencing transcription factor (REST) and heat shock protein 90 as new therapeutic target against Huntington’s disease. PLoS One.

[36] Calvo-Vidal M.N., Zamponi N., Krumsiek J., Stockslager M.A., Revuelta M.V., Phillip J.M., Marullo R., Tikhonova E., Kotlov N., Patel J. (2021). Oncogenic HSP90 Facilitates Metabolic Alterations in Aggressive B-cell Lymphomas. Cancer Res..

[37] Partridge J.R., Lavery L.A., Elnatan D., Naber N., Cooke R., Agard D.A. (2014). A novel N-terminal extension in mitochondrial TRAP1 serves as a thermal regulator of chaperone activity. eLife.

[38] Elnatan D., Betegon M., Liu Y., Ramelot T., Kennedy M.A., Agard D.A. (2017). Symmetry broken and rebroken during the ATP hydrolysis cycle of the mitochondrial Hsp90 TRAP1. eLife.

[39] Jumper J., Evans R., Pritzel A., Green T., Figurnov M., Ronneberger O., Tunyasuvunakool K., Bates R., Žídek A., Potapenko A. (2021). Highly accurate protein structure prediction with AlphaFold. Nature.

[40] Varadi M., Bertoni D., Magana P., Paramval U., Pidruchna I., Radhakrishnan M., Tsenkov M., Nair S., Mirdita M., Yeo J. (2024). AlphaFold Protein Structure Database in 2024: providing structure coverage for over 214 million protein sequences. Nucleic Acids Res..

[41] Kang B.H., Siegelin M.D., Plescia J., Raskett C.M., Garlick D.S., Dohi T., Lian J.B., Stein G.S., Languino L.R., Altieri D.C. (2010). Preclinical Characterization of Mitochondria-Targeted Small Molecule Hsp90 Inhibitors, Gamitrinibs, in Advanced Prostate Cancer. Clin. Cancer Res..

[42] Pang Z., Lu Y., Zhou G., Hui F., Xu L., Viau C., Spigelman A.F., MacDonald P.E., Wishart D.S., Li S. (2024). MetaboAnalyst 6.0: towards a unified platform for metabolomics data processing, analysis and interpretation. Nucleic Acids Res..

[43] Kim S., Backe S.J., Wengert L.A., Johnson A.E., Isakov R.V., Bratslavsky M.S., Woodford M.R. (2022). O-GlcNAcylation suppresses TRAP1 activity and promotes mitochondrial respiration. Cell Stress Chaperones.

[44] Falsone S.F., Gesslbauer B., Tirk F., Piccinini A.-M., Kungl A.J. (2005). A proteomic snapshot of the human heat shock protein 90 interactome. FEBS Lett..

[45] Shen G., Liu S., Cao Y., Chen Z., Wang G., Yu L., Sun L., Ran Y. (2025). HSP90 co-regulates the formation and nuclear distribution of the glycolytic output complex to promote resistance and poor prognosis in gastric cancer patients. J. Transl. Med..

[46] Subramanian C., Hohenberger K.K., Zuo A., Cousineau E., Blagg B., Cohen M. (2025). C-Terminal Hsp90 Inhibitors Overcome MEK and BRAF Inhibitor Resistance in Melanoma. J. Cell. Mol. Med..

[47] Subramanian C., Gorney R., Wang T., Ge D., Zhang N., Zuo A., Blagg B.S.J., Cohen M.S. (2021). A novel heat shock protein inhibitor KU757 with efficacy in lenvatinib-resistant follicular thyroid cancer cells overcomes up-regulated glycolysis in drug-resistant cells in vitro. Surgery.

[48] Heck A.L., Mishra S., Prenzel T., Feulner L., Achhammer E., Särchen V., Blagg B.S.J., Schneider-Brachert W., Schütze S., Fritsch J. (2021). Selective HSP90β inhibition results in TNF and TRAIL mediated HIF1α degradation. Immunobiology.

[49] Merfeld T., Peng S., Keegan B.M., Crowley V.M., Brackett C.M., Gutierrez A., McCann N.R., Reynolds T.S., Rhodes M.C., Byrd K.M. (2023). Elucidation of novel TRAP1-Selective inhibitors that regulate mitochondrial processes. Eur. J. Med. Chem..

[50] Mishra S.J., Khandelwal A., Banerjee M., Balch M., Peng S., Davis R.E., Merfeld T., Munthali V., Deng J., Matts R.L. (2021). Selective Inhibition of the Hsp90α Isoform. Angew. Chem. Int. Ed..

[51] Khandelwal A., Kent C.N., Balch M., Peng S., Mishra S.J., Deng J., Day V.W., Liu W., Subramanian C., Cohen M. (2018). Structure-guided design of an Hsp90β N-terminal isoform-selective inhibitor. Nat. Commun..

[52] Borst P. (2020). The malate–aspartate shuttle (Borst cycle): How it started and developed into a major metabolic pathway. IUBMB Life.

[53] Joshi A., Dai L., Liu Y., Lee J., Ghahhari N.M., Segala G., Beebe K., Jenkins L.M., Lyons G.C., Bernasconi L. (2020). The mitochondrial HSP90 paralog TRAP1 forms an OXPHOS-regulated tetramer and is involved in mitochondrial metabolic homeostasis. BMC Biol..

[54] Backe S.J., Sager R.A., Woodford M.R., Makedon A.M., Mollapour M. (2020). Post-translational modifications of Hsp90 and translating the chaperone code. J. Biol. Chem..

[55] Felipe Perez R., Mochi G., Khan A., Woodford M. (2024). Mitochondrial Chaperone Code: Just warming up. Cell Stress Chaperones.

[56] Rizza S., Montagna C., Cardaci S., Maiani E., Di Giacomo G., Sanchez-Quiles V., Blagoev B., Rasola A., De Zio D., Stamler J.S. (2016). S-nitrosylation of the Mitochondrial Chaperone TRAP1 Sensitizes Hepatocellular Carcinoma Cells to Inhibitors of Succinate Dehydrogenase. Cancer Res..

[57] Faienza F., Lambrughi M., Rizza S., Pecorari C., Giglio P., Salamanca Viloria J., Allega M.F., Chiappetta G., Vinh J., Pacello F. (2020). S-nitrosylation affects TRAP1 structure and ATPase activity and modulates cell response to apoptotic stimuli. Biochem. Pharmacol..

[58] Barrott J.J., Haystead T.A.J. (2013). Hsp90, an unlikely ally in the war on cancer. FEBS J..

[59] Bhattacharya K., Picard D. (2021). The Hsp70–Hsp90 go-between Hop/Stip1/Sti1 is a proteostatic switch and may be a drug target in cancer and neurodegeneration. Cell. Mol. Life Sci..

[60] Shelton L.B., Koren J., Blair L.J. (2017). Imbalances in the Hsp90 Chaperone Machinery: Implications for Tauopathies. Front. Neurosci..

[61] Youker R.T., Walsh P., Beilharz T., Lithgow T., Brodsky J.L. (2004). Distinct Roles for the Hsp40 and Hsp90 Molecular Chaperones during Cystic Fibrosis Transmembrane Conductance Regulator Degradation in Yeast. MBoC.

[62] Neckers L., Workman P. (2012). Hsp90 Molecular Chaperone Inhibitors: Are We There Yet?. Clin. Cancer Res..

[63] Henry O., Jolicoeur M., Kamen A. (2011). Unraveling the metabolism of HEK-293 cells using lactate isotopomer analysis. Bioprocess Biosyst. Eng..

[64] Marin-Valencia I., Cho S.K., Rakheja D., Hatanpaa K.J., Kapur P., Mashimo T., Jindal A., Vemireddy V., Good L.B., Raisanen J. (2012). Glucose metabolism via the pentose phosphate pathway, glycolysis and Krebs cycle in an orthotopic mouse model of human brain tumors. NMR Biomed..

[65] Patra K.C., Hay N. (2014). The pentose phosphate pathway and cancer. Trends Biochem. Sci..

[66] Abdouh M., Zhou S., Arena V., Arena M., Lazaris A., Onerheim R., Metrakos P., Arena G.O. (2014). Transfer of malignant trait to immortalized human cells following exposure to human cancer serum. Journal of Experimental \& Clinical Cancer Research.

[67] Perez-Riverol Y., Bandla C., Kundu D.J., Kamatchinathan S., Bai J., Hewapathirana S., John N.S., Prakash A., Walzer M., Wang S. (2025). The PRIDE database at 20 years: 2025 update. Nucleic Acids Res..

[68] Schneider C.A., Rasband W.S., Eliceiri K.W. (2012). NIH Image to ImageJ: 25 years of image analysis. Nat. Methods.

[69] Shannon P., Markiel A., Ozier O., Baliga N.S., Wang J.T., Ramage D., Amin N., Schwikowski B., Ideker T. (2003). Cytoscape: A Software Environment for Integrated Models of Biomolecular Interaction Networks. Genome Res..

[70] Szklarczyk D., Kirsch R., Koutrouli M., Nastou K., Mehryary F., Hachilif R., Gable A.L., Fang T., Doncheva N.T., Pyysalo S. (2023). The STRING database in 2023: protein-protein association networks and functional enrichment analyses for any sequenced genome of interest. Nucleic Acids Res..

[71] Ge S.X., Jung D., Yao R. (2020). ShinyGO: a graphical gene-set enrichment tool for animals and plants. Bioinformatics.

[72] Bhattacharya K., Maiti S., Zahoran S., Weidenauer L., Hany D., Wider D., Bernasconi L., Quadroni M., Collart M., Picard D. (2022). Translational reprogramming in response to accumulating stressors ensures critical threshold levels of Hsp90 for mammalian life. Nat. Commun..

[73] Wang X., Middleton F.A., Tawil R., Chen X.J. (2022). Cytosolic adaptation to mitochondria-induced proteostatic stress causes progressive muscle wasting. iScience.

[74] Luo W., Brouwer C. (2013). Pathview: an R/Bioconductor package for pathway-based data integration and visualization. Bioinformatics.

