# Supplementary material for: Coordinated regulation of the metaboproteome by Hsp90 chaperones controls metabolic plasticity
Source: iScience. 2026 Sep 10;29(10):116105. doi: 10.1016/j.isci.2026.116105 (PMC13586520; doi:10.1016/j.isci.2026.116105)
Supplement: Supplementary file 1 [file mmc1.pdf]

iScience, Volume *29*

Supplemental information

Coordinated regulation of the metaboproteome by Hsp90 chaperones controls metabolic plasticity

R. Felipe Perez, Gianna L. Mochi, Julia K. Burkacki, Victoria Zoccoli-Rodriguez, Landon D. Arcadi, Sarah J. Backe, Joel R. Wilmore, and Mark R. Woodford

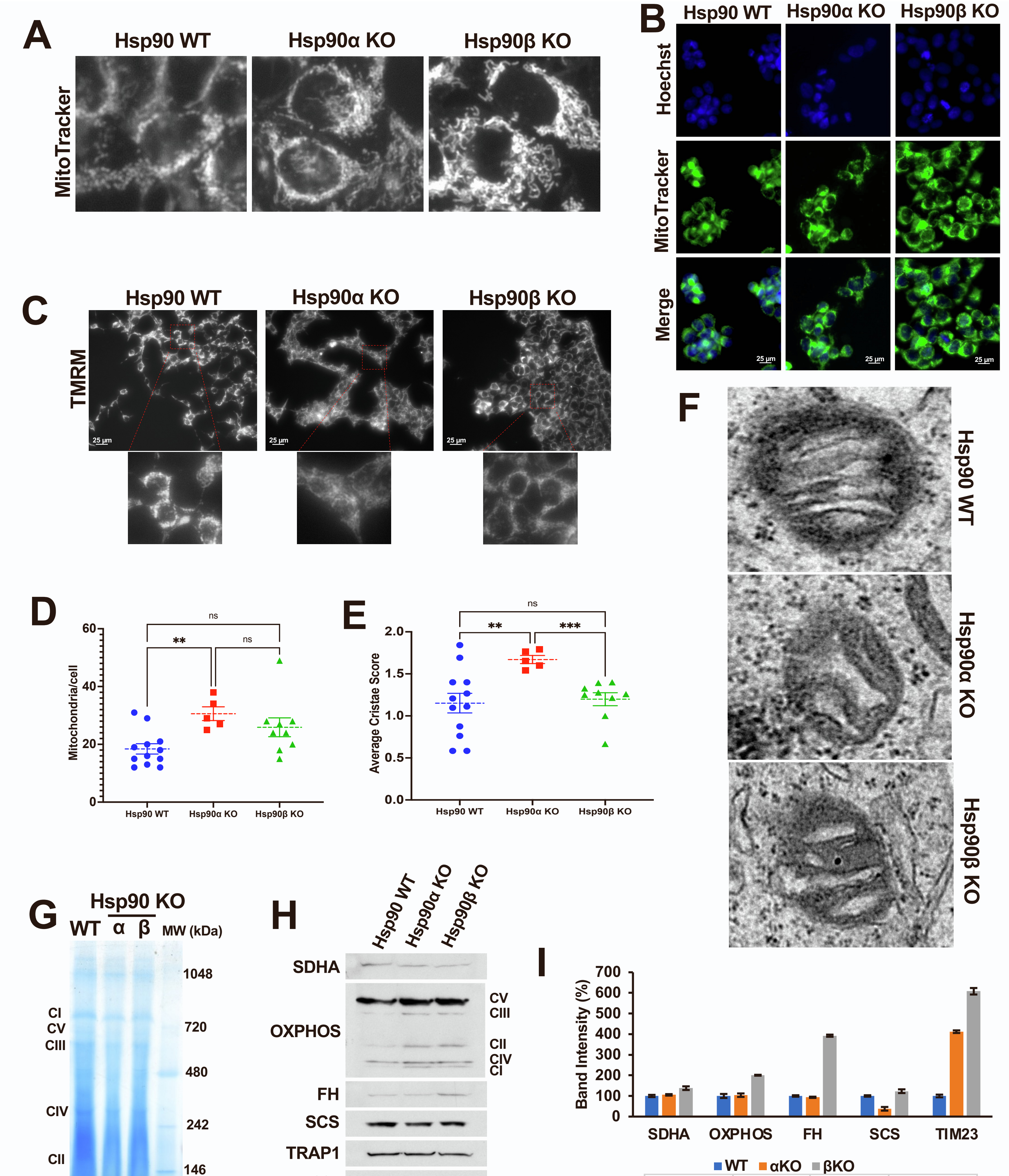

	WT v αKO	WT v βKO	αKO v βKO
SDHA	*	**	*
OXPHOS	NS	**	***
FH	*	***	***
SCS	**	*	**
TIM23	***	**	*

Figure S1

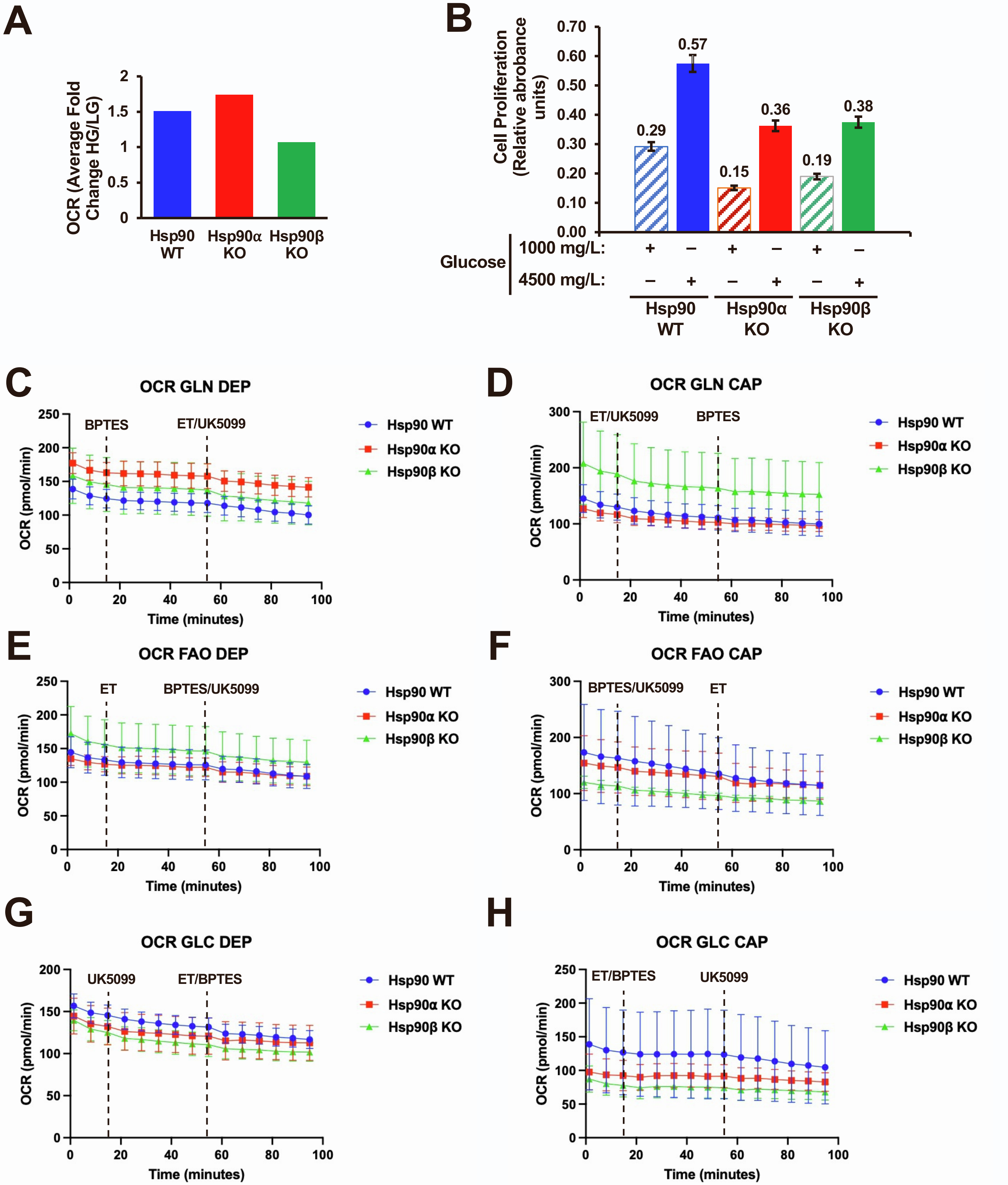

A
OCR (Average Fold Change HG/LG)
2
1.5
1
0.5
0
Hsp90 WT
Hsp90α KO
Hsp90β KO
B
Cell Proliferation (Relative abrobance units)
0.70
0.60
0.50
0.40
0.30
0.20
0.10
0.00
0.29
0.57
0.15
0.36
0.19
0.38
Glucose
1000 mg/L: + − + − + −
4500 mg/L: − + − + − +
Hsp90 WT
Hsp90α KO
Hsp90β KO
C
OCR GLN DEP
BPTES
ET/UK5099
OCR (pmol/min)
Time (minutes)
Hsp90 WT
Hsp90α KO
Hsp90β KO
D
OCR GLN CAP
ET/UK5099
BPTES
OCR (pmol/min)
Time (minutes)
Hsp90 WT
Hsp90α KO
Hsp90β KO
E
OCR FAO DEP
ET
BPTES/UK5099
OCR (pmol/min)
Time (minutes)
Hsp90 WT
Hsp90α KO
Hsp90β KO
F
OCR FAO CAP
BPTES/UK5099
ET
OCR (pmol/min)
Time (minutes)
Hsp90 WT
Hsp90α KO
Hsp90β KO
G
OCR GLC DEP
UK5099
ET/BPTES
OCR (pmol/min)
Time (minutes)
Hsp90 WT
Hsp90α KO
Hsp90β KO
H
OCR GLC CAP
ET/BPTES
UK5099
OCR (pmol/min)
Time (minutes)
Hsp90 WT
Hsp90α KO
Hsp90β KO

Figure S2

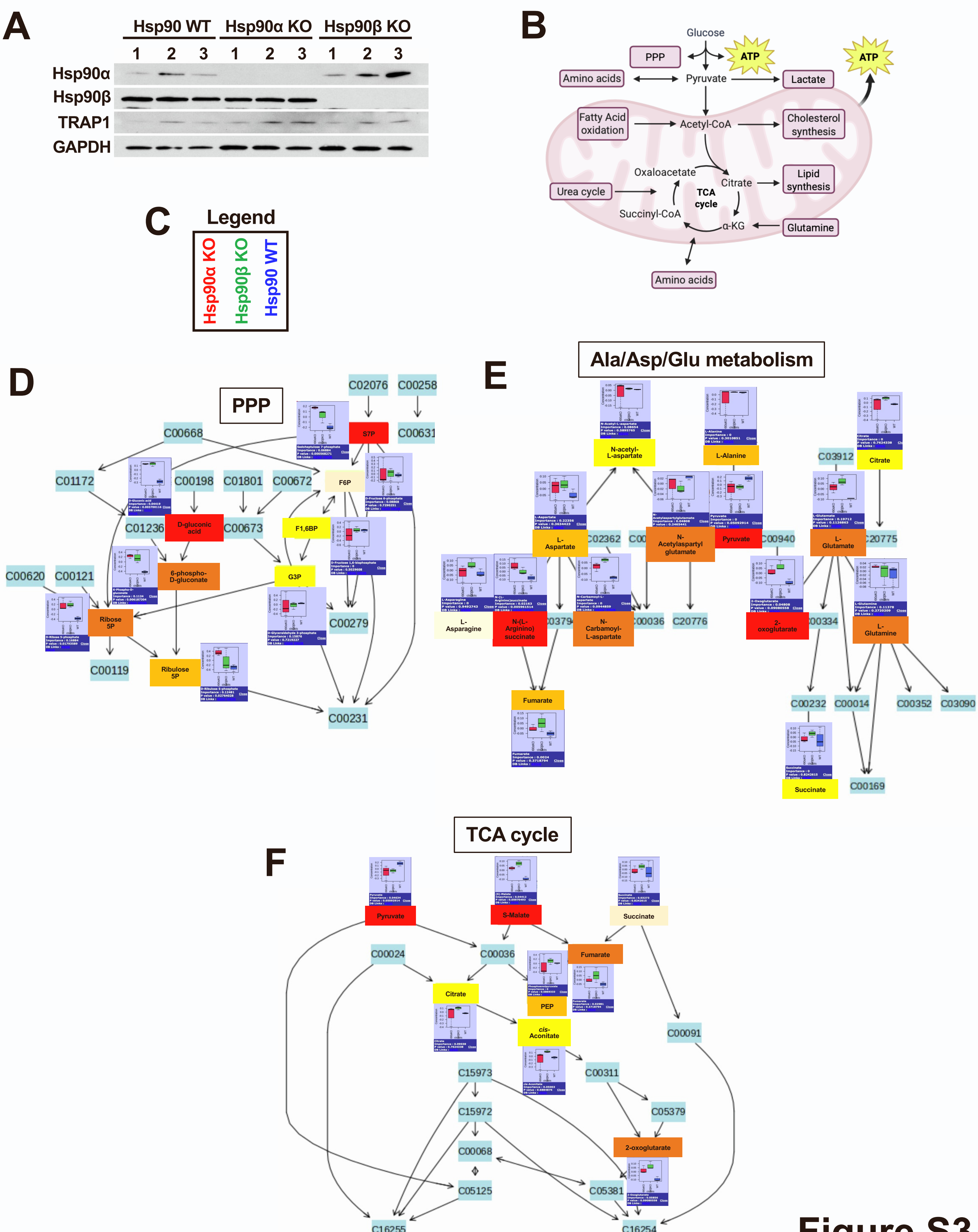

A
Hsp90 WT
Hsp90α KO
Hsp90β KO
1 2 3 1 2 3 1 2 3
Hsp90α
Hsp90β
TRAP1
GAPDH
B
Glucose
PPP
ATP
Amino acids
Pyruvate
Lactate
ATP
Fatty Acid oxidation
Acetyl-CoA
Cholesterol synthesis
Oxaloacetate
Citrate
Lipid synthesis
Urea cycle
TCA cycle
Succinyl-CoA
α-KG
Glutamine
Amino acids
C
Legend
Hsp90α KO
Hsp90β KO
Hsp90 WT
D
PPP
C02076
C00258
C00668
S7P
C00631
C01172
C00198
C01801
C00672
F6P
C01236
D-gluconic acid
C00673
F1,6BP
C00620
C00121
6-phospho-D-gluconate
G3P
Ribose 5P
C00279
C00119
Ribulose 5P
C00231
E
Ala/Asp/Glu metabolism
N-acetyl-L-aspartate
L-Alanine
C03912
Citrate
L-Aspartate
C02362
Acetylaspartyl glutamate
Pyruvate
C00940
L-Glutamate
C20775
L-Asparagine
N-(L-Arginino) succinate
N-Carbamoyl-L-aspartate
C00036
C20776
2-oxoglutarate
C00334
L-Glutamine
Fumarate
C00232
C00014
C00352
C03090
Succinate
C00169
F
TCA cycle
Pyruvate
S-Malate
Succinate
C00024
C00036
Fumarate
Citrate
PEP
cis-Aconitate
C00091
C15973
C00311
C15972
C05379
2-oxoglutarate
C00068
C05125
C05381
C16255
C16254

Figure S3

Figure S4

A

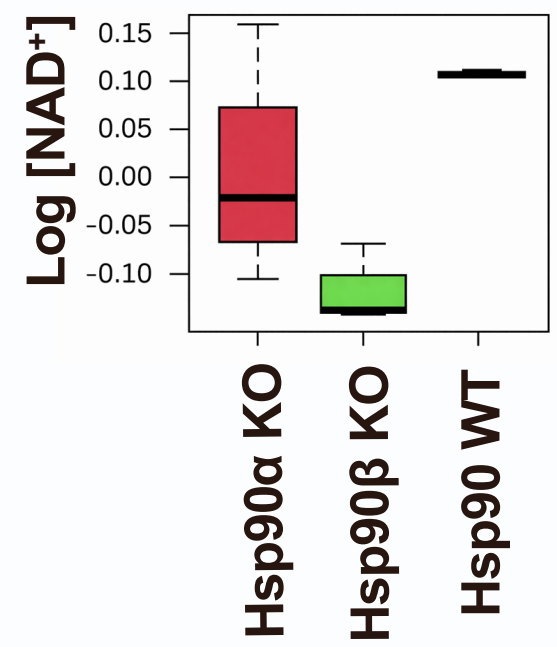

B

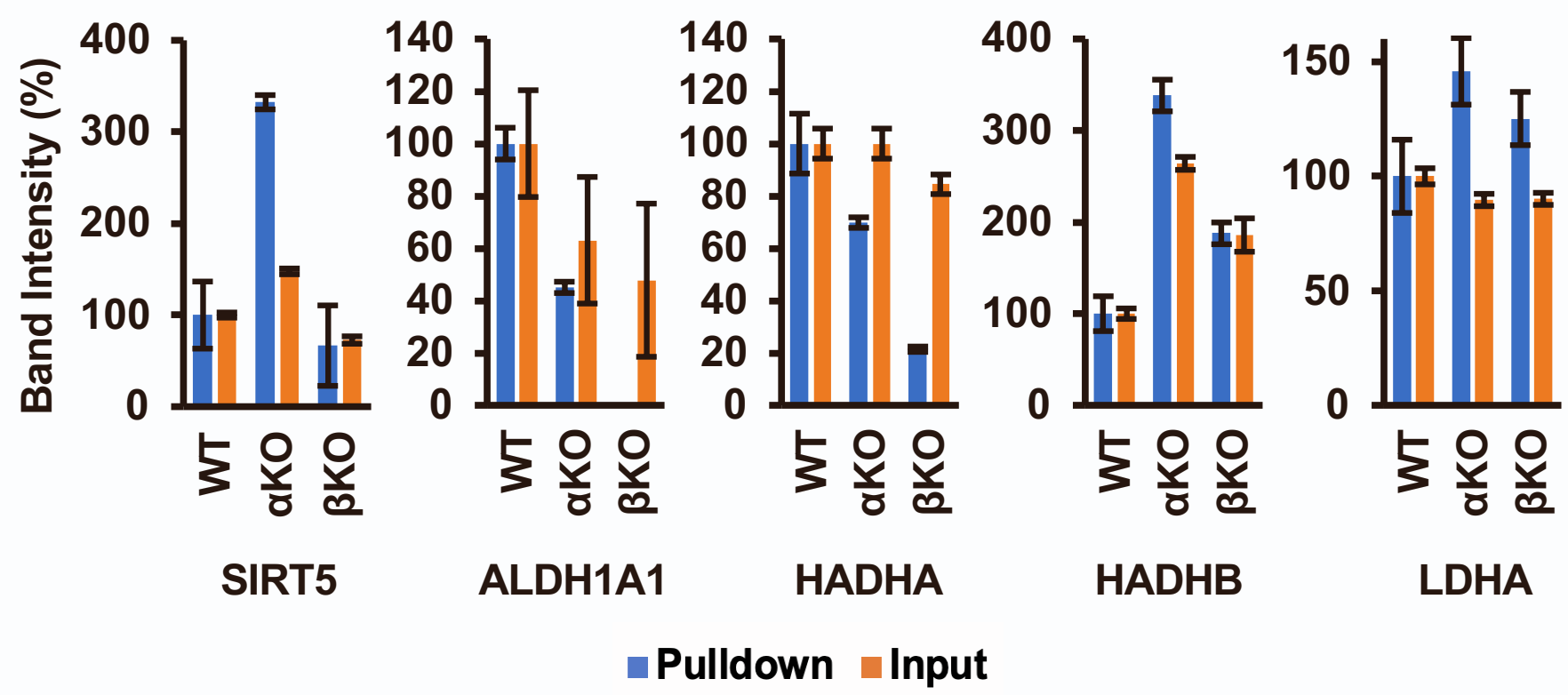

	SIRT5		ALDH1A1		HADHA		HADHB		LDHA	
	Pulldown	Input	Pulldown	Input	Pulldown	Input	Pulldown	Input	Pulldown	Input
WT v αKO	***	**	**	**	*	*	**	**	**	NS
WT v βKO	**	**	***	**	**	*	*	*	*	**
αKO v βKO	***	***	****	**	****	*	*	**	NS	*

Figure S5

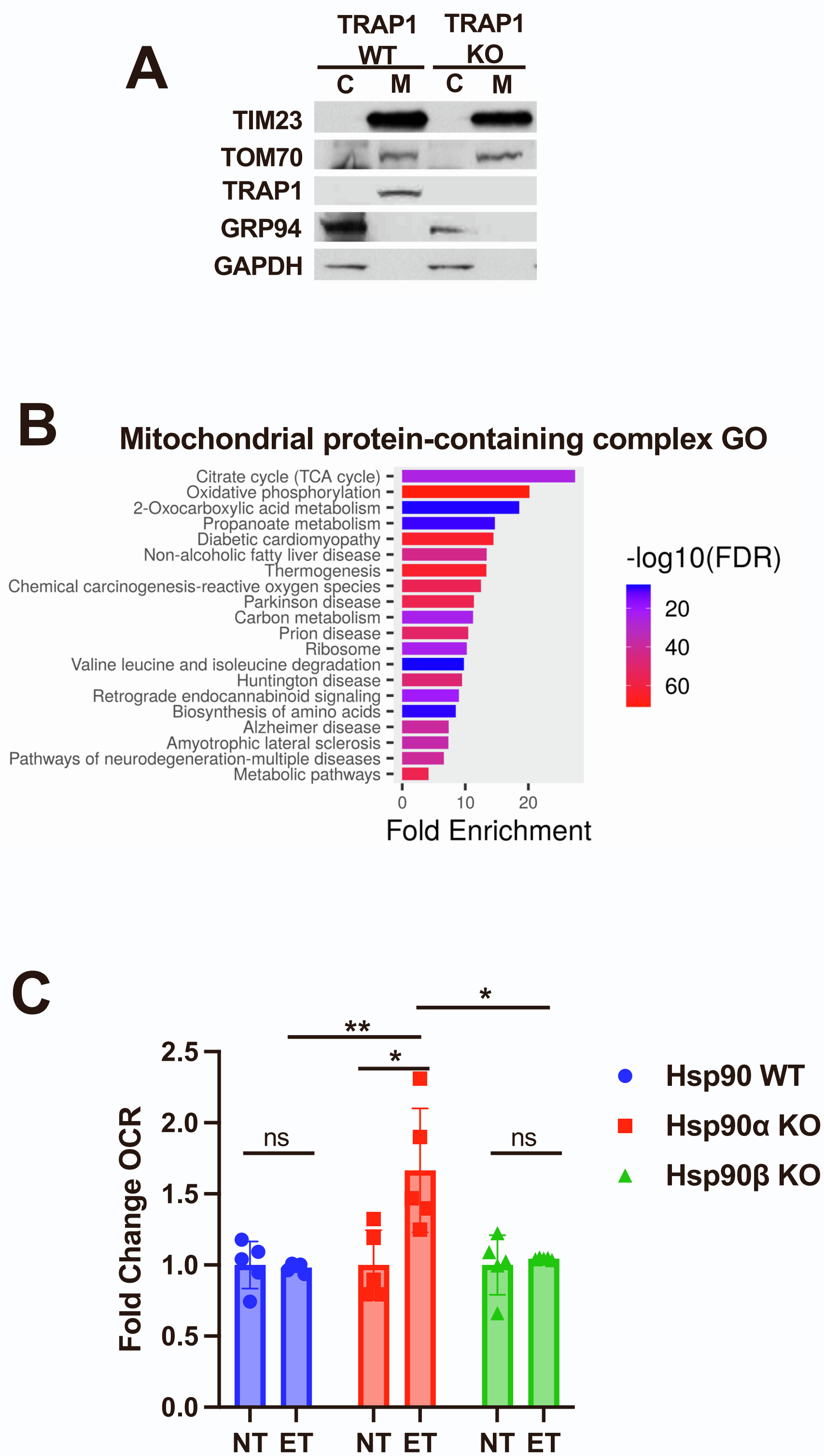
A
TRAP1 WT
TRAP1 KO
C M C M
TIM23
TOM70
TRAP1
GRP94
GAPDH
B
Mitochondrial protein-containing complex GO
Citrate cycle (TCA cycle)
Oxidative phosphorylation
2-Oxocarboxylic acid metabolism
Propanoate metabolism
Diabetic cardiomyopathy
Non-alcoholic fatty liver disease
Thermogenesis
Chemical carcinogenesis-reactive oxygen species
Parkinson disease
Carbon metabolism
Prion disease
Ribosome
Valine leucine and isoleucine degradation
Huntington disease
Retrograde endocannabinoid signaling
Biosynthesis of amino acids
Alzheimer disease
Amyotrophic lateral sclerosis
Pathways of neurodegeneration-multiple diseases
Metabolic pathways
0 10 20
Fold Enrichment
-log10(FDR)
20
40
60
C
Fold Change OCR
0.0 0.5 1.0 1.5 2.0 2.5
ns
**
*
*
ns
NT ET NT ET NT ET
Hsp90 WT
Hsp90α KO
Hsp90β KO

Figure S6

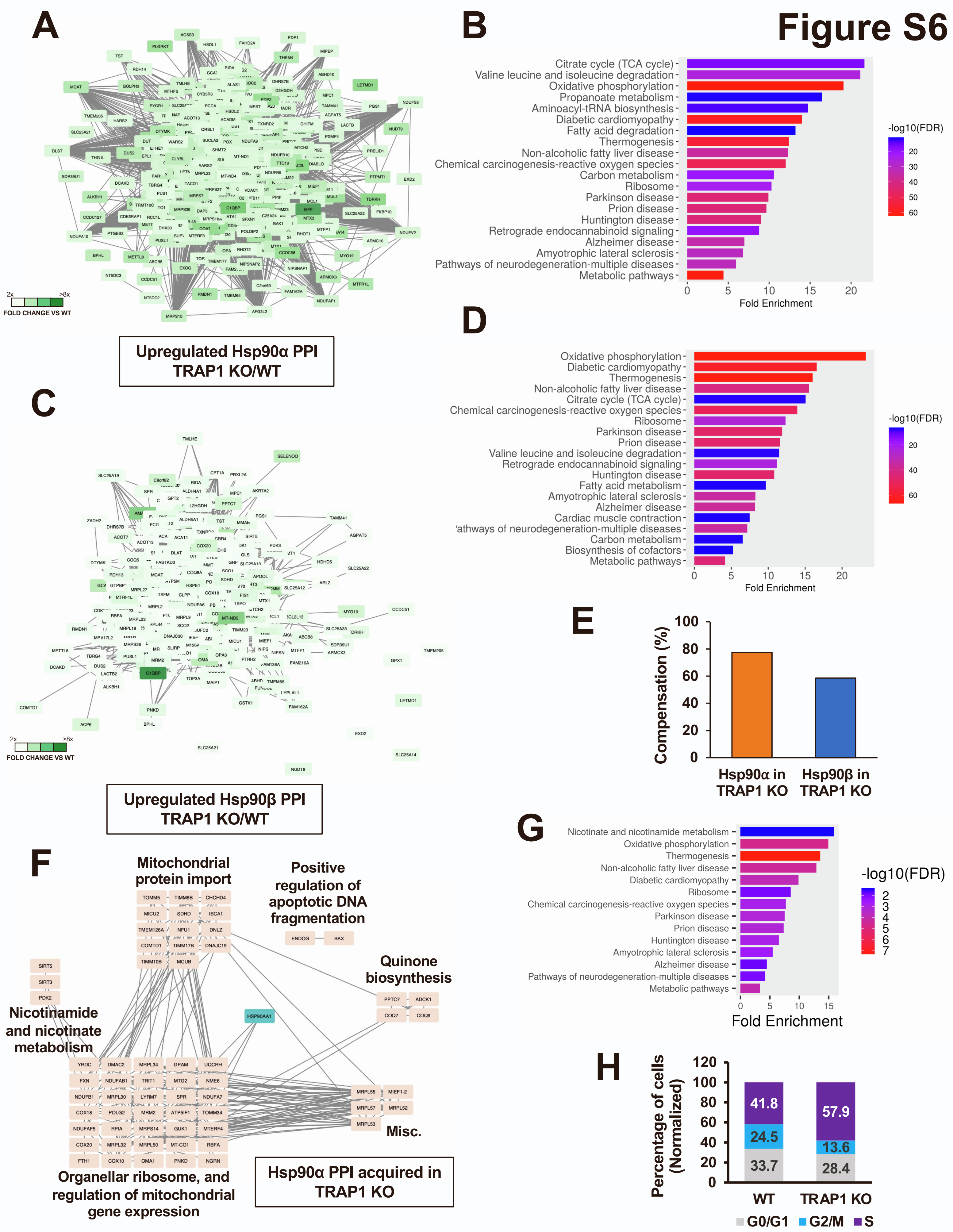
A
2x
>8x
FOLD CHANGE VS WT
Upregulated Hsp90α PPI TRAP1 KO/WT
B
Citrate cycle (TCA cycle)
Valine leucine and isoleucine degradation
Oxidative phosphorylation
Propanoate metabolism
Aminoacyl-tRNA biosynthesis
Diabetic cardiomyopathy
Fatty acid degradation
Thermogenesis
Non-alcoholic fatty liver disease
Chemical carcinogenesis-reactive oxygen species
Carbon metabolism
Ribosome
Parkinson disease
Prion disease
Huntington disease
Retrograde endocannabinoid signaling
Alzheimer disease
Amyotrophic lateral sclerosis
Pathways of neurodegeneration-multiple diseases
Metabolic pathways
0 5 10 15 20
Fold Enrichment
-log10(FDR)
20 30 40 50 60
C
2x
>8x
FOLD CHANGE VS WT
Upregulated Hsp90β PPI TRAP1 KO/WT
D
Oxidative phosphorylation
Diabetic cardiomyopathy
Thermogenesis
Non-alcoholic fatty liver disease
Citrate cycle (TCA cycle)
Chemical carcinogenesis-reactive oxygen species
Ribosome
Parkinson disease
Prion disease
Valine leucine and isoleucine degradation
Retrograde endocannabinoid signaling
Huntington disease
Fatty acid metabolism
Amyotrophic lateral sclerosis
Alzheimer disease
Cardiac muscle contraction
'athways of neurodegeneration-multiple diseases
Carbon metabolism
Biosynthesis of cofactors
Metabolic pathways
0 5 10 15 20
Fold Enrichment
-log10(FDR)
20 40 60
E
Compensation (%)
0 20 40 60 80 100
Hsp90α in TRAP1 KO
Hsp90β in TRAP1 KO
F
Mitochondrial protein import
TOMM5 TIMM8B CHCHD4
MICU2 SDHD ISCA1
TMEM126A NFU1 DNLZ
COMTD1 TIMM17B DNAJC19
TIMM10B MCUB
Positive regulation of apoptotic DNA fragmentation
ENDOG BAX
SIRT5 SIRT3 PDK2
Nicotinamide and nicotinate metabolism
HSP90AA1
Quinone biosynthesis
PPTC7 ADCK1 COQ7 COQ9
YRDC DMAC2 MRPL34 GPAM UQCRH
FXN NDUFAB1 TRIT1 MTG2 NME6
NDUFB1 MRPL30 LYRM7 SPR NDUFA7
COX18 POLG2 MRM2 ATP5IF1 TOMM34
NDUFAF5 RPIA MRPS14 GUK1 MTERF4
COX20 MRPL32 MRPL50 MT-CO1 RBFA
FTH1 COX10 OMA1 PNKD NGRN
MRPL55 MIEF1-2
MRPL57 MRPL52
MRPL53
Misc.
Organellar ribosome, and regulation of mitochondrial gene expression
Hsp90α PPI acquired in TRAP1 KO
G
Nicotinate and nicotinamide metabolism
Oxidative phosphorylation
Thermogenesis
Non-alcoholic fatty liver disease
Diabetic cardiomyopathy
Ribosome
Chemical carcinogenesis-reactive oxygen species
Parkinson disease
Prion disease
Huntington disease
Amyotrophic lateral sclerosis
Alzheimer disease
Pathways of neurodegeneration-multiple diseases
Metabolic pathways
0 5 10 15
Fold Enrichment
-log10(FDR)
2 3 4 5 6 7
H
Percentage of cells (Normalized)
0 20 40 60 80 100 120
41.8
24.5
33.7
57.9
13.6
28.4
WT
TRAP1 KO
G0/G1 G2/M S

Figure S7

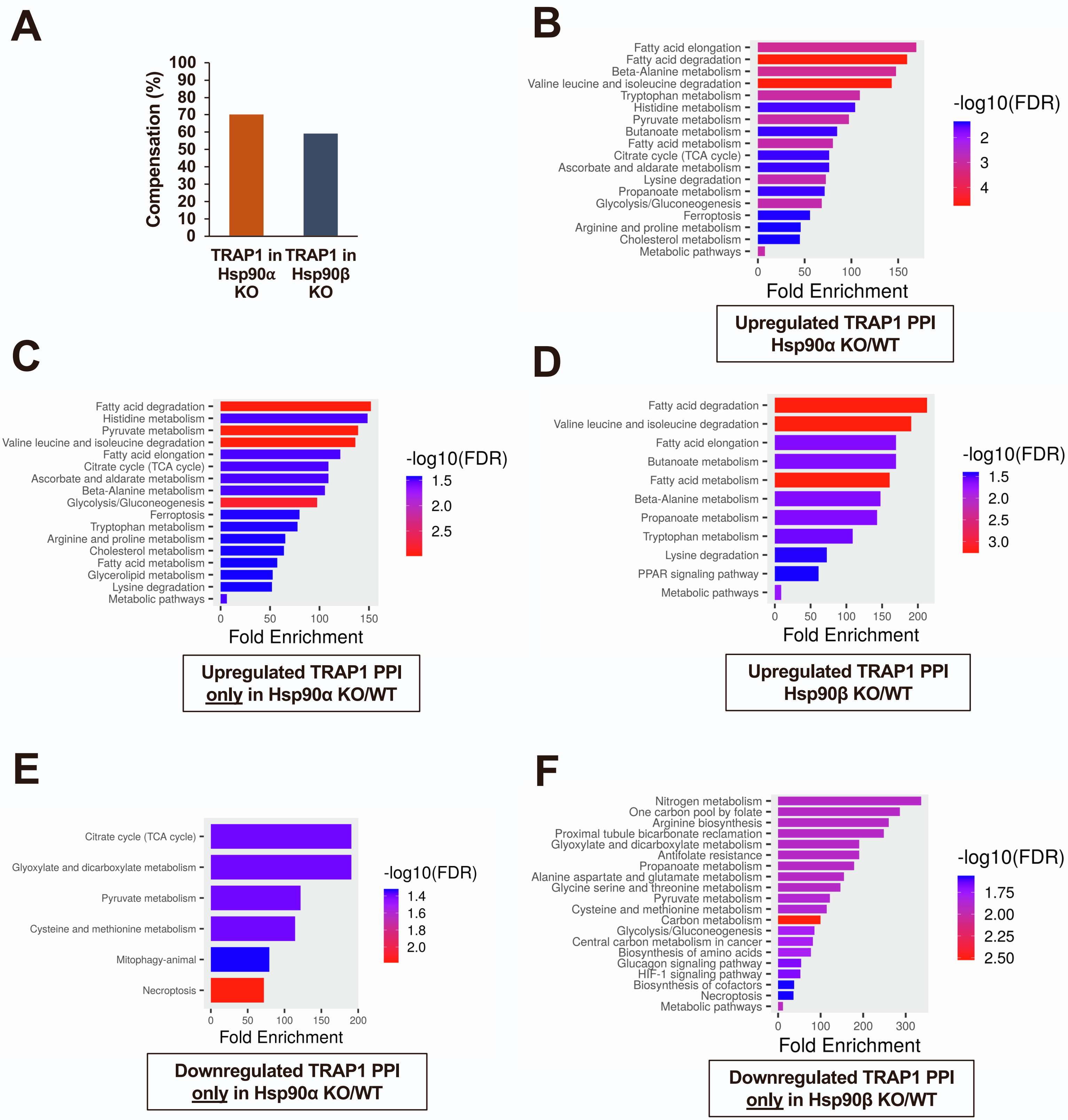

A
Compensation (%)
100
90
80
70
60
50
40
30
20
10
0
TRAP1 in Hsp90α KO
TRAP1 in Hsp90β KO
B
Fatty acid elongation
Fatty acid degradation
Beta-Alanine metabolism
Valine leucine and isoleucine degradation
Tryptophan metabolism
Histidine metabolism
Pyruvate metabolism
Butanoate metabolism
Fatty acid metabolism
Citrate cycle (TCA cycle)
Ascorbate and aldarate metabolism
Lysine degradation
Propanoate metabolism
Glycolysis/Gluconeogenesis
Ferroptosis
Arginine and proline metabolism
Cholesterol metabolism
Metabolic pathways
0
50
100
150
Fold Enrichment
-log10(FDR)
2
3
4
Upregulated TRAP1 PPI Hsp90α KO/WT
C
Fatty acid degradation
Histidine metabolism
Pyruvate metabolism
Valine leucine and isoleucine degradation
Fatty acid elongation
Citrate cycle (TCA cycle)
Ascorbate and aldarate metabolism
Beta-Alanine metabolism
Glycolysis/Gluconeogenesis
Ferroptosis
Tryptophan metabolism
Arginine and proline metabolism
Cholesterol metabolism
Fatty acid metabolism
Glycerolipid metabolism
Lysine degradation
Metabolic pathways
0
50
100
150
Fold Enrichment
-log10(FDR)
1.5
2.0
2.5
Upregulated TRAP1 PPI only in Hsp90α KO/WT
D
Fatty acid degradation
Valine leucine and isoleucine degradation
Fatty acid elongation
Butanoate metabolism
Fatty acid metabolism
Beta-Alanine metabolism
Propanoate metabolism
Tryptophan metabolism
Lysine degradation
PPAR signaling pathway
Metabolic pathways
0
50
100
150
200
Fold Enrichment
-log10(FDR)
1.5
2.0
2.5
3.0
Upregulated TRAP1 PPI Hsp90β KO/WT
E
Citrate cycle (TCA cycle)
Glyoxylate and dicarboxylate metabolism
Pyruvate metabolism
Cysteine and methionine metabolism
Mitophagy-animal
Necroptosis
0
50
100
150
200
Fold Enrichment
-log10(FDR)
1.4
1.6
1.8
2.0
Downregulated TRAP1 PPI only in Hsp90α KO/WT
F
Nitrogen metabolism
One carbon pool by folate
Arginine biosynthesis
Proximal tubule bicarbonate reclamation
Glyoxylate and dicarboxylate metabolism
Antifolate resistance
Propanoate metabolism
Alanine aspartate and glutamate metabolism
Glycine serine and threonine metabolism
Pyruvate metabolism
Cysteine and methionine metabolism
Carbon metabolism
Glycolysis/Gluconeogenesis
Central carbon metabolism in cancer
Biosynthesis of amino acids
Glucagon signaling pathway
HIF-1 signaling pathway
Biosynthesis of cofactors
Necroptosis
Metabolic pathways
0
100
200
300
Fold Enrichment
-log10(FDR)
1.75
2.00
2.25
2.50
Downregulated TRAP1 PPI only in Hsp90β KO/WT

Figure S8

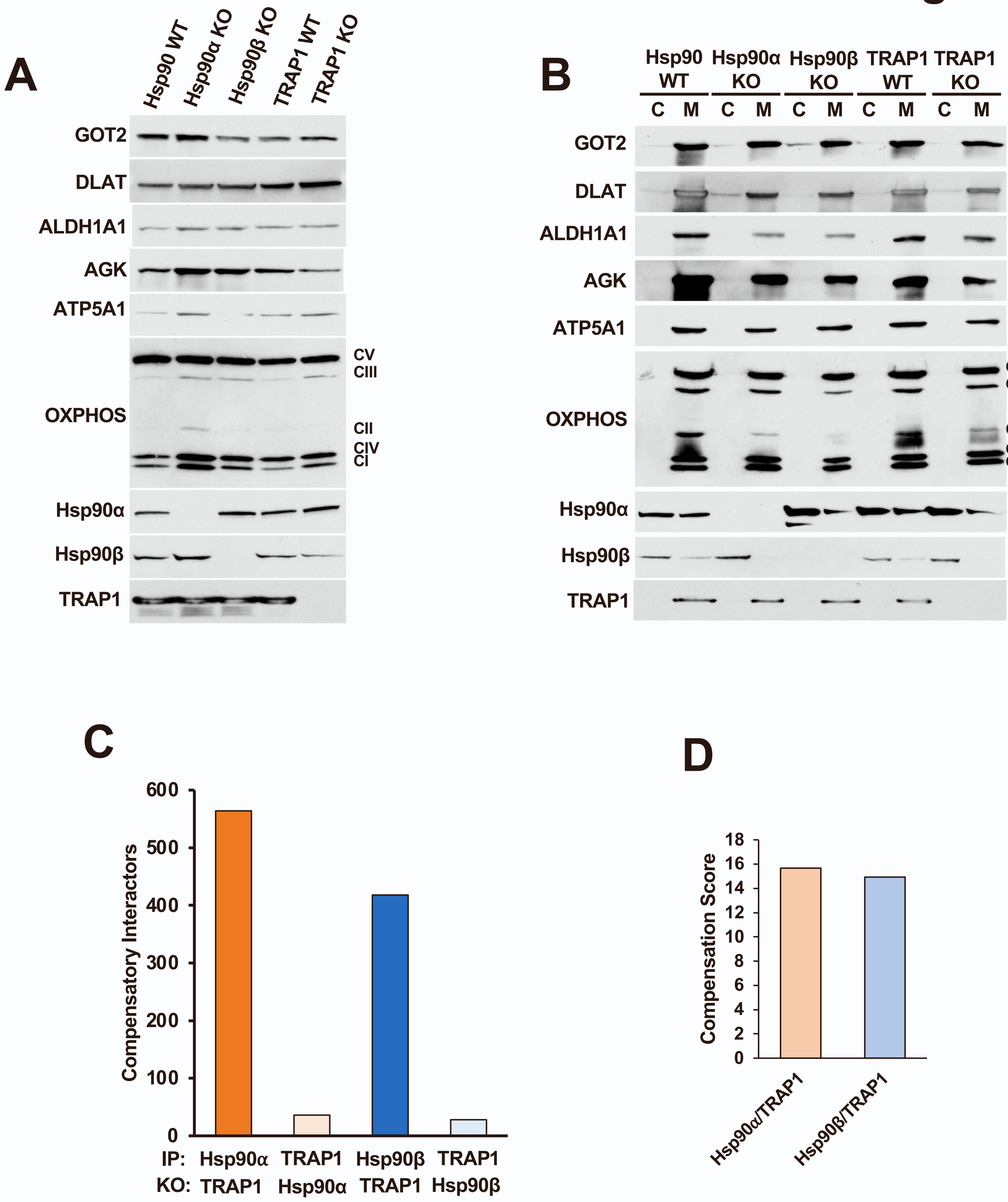
A
Hsp90 WT
Hsp90α KO
Hsp90β KO
TRAP1 WT
TRAP1 KO
GOT2
DLAT
ALDH1A1
AGK
ATP5A1
OXPHOS
CV
CIII
CII
CIV
CI
Hsp90α
Hsp90β
TRAP1
B
Hsp90 WT
Hsp90α KO
Hsp90β KO
TRAP1 WT
TRAP1 KO
C M C M C M C M C M
GOT2
DLAT
ALDH1A1
AGK
ATP5A1
OXPHOS
CV
CIII
CII
CIV
CI
Hsp90α
Hsp90β
TRAP1
C
Compensatory Interactors
0
100
200
300
400
500
600
IP: Hsp90α TRAP1 Hsp90β TRAP1
KO: TRAP1 Hsp90α TRAP1 Hsp90β
D
Compensation Score
0
2
4
6
8
10
12
14
16
18
Hsp90α/TRAP1
Hsp90β/TRAP1

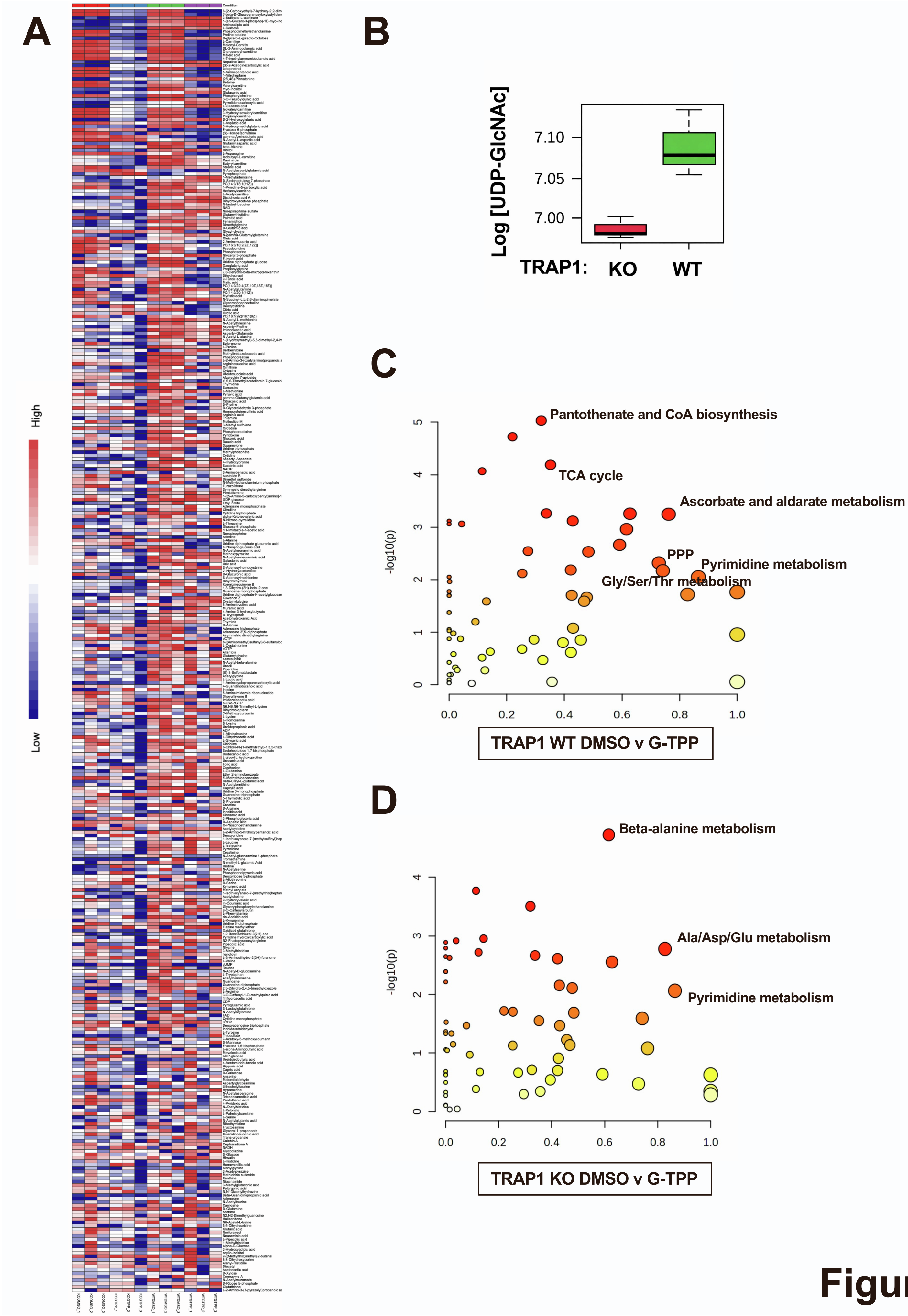
A
High
Low
B
Log [UDP-GlcNAc]
7.10
7.05
7.00
TRAP1:
KO
WT
C
Pantothenate and CoA biosynthesis
TCA cycle
Ascorbate and aldarate metabolism
PPP
Pyrimidine metabolism
Gly/Ser/Thr metabolism
-log10(p)
0
1
2
3
4
5
0.0
0.2
0.4
0.6
0.8
1.0
TRAP1 WT DMSO v G-TPP
D
Beta-alanine metabolism
Ala/Asp/Glu metabolism
Pyrimidine metabolism
-log10(p)
0
1
2
3
4
0.0
0.2
0.4
0.6
0.8
1.0
TRAP1 KO DMSO v G-TPP

Figure S9

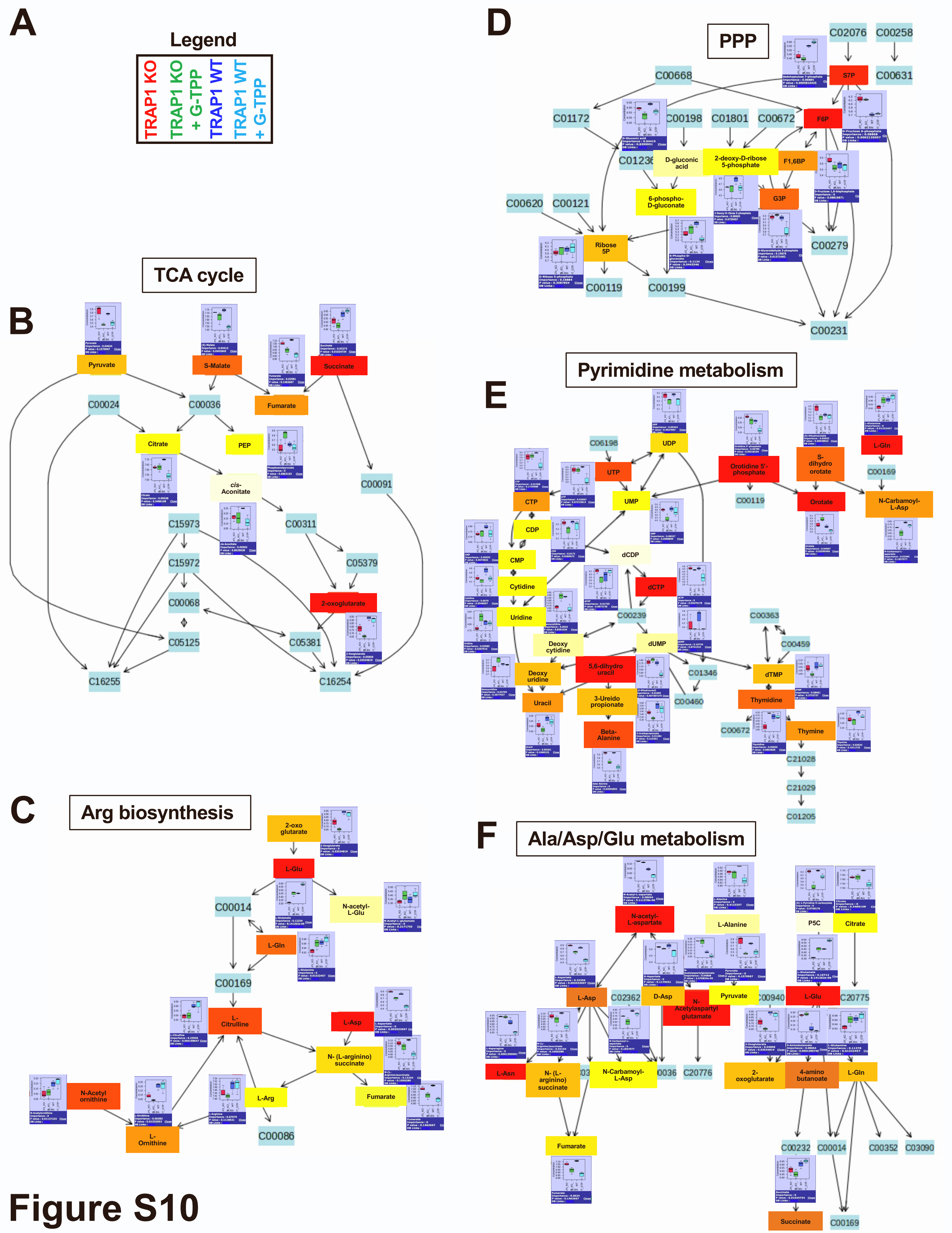

A
Legend
TRAP1 KO
TRAP1 KO + G-TPP
TRAP1 WT
TRAP1 WT + G-TPP
TCA cycle
B
Pyruvate
S-Malate
Succinate
Fumarate
C00024
C00036
Citrate
PEP
cis-Aconitate
C00091
C15973
C00311
C15972
C05379
C00068
2-oxoglutarate
C05125
C05381
C16255
C16254
C
Arg biosynthesis
2-oxo glutarate
L-Glu
C00014
N-acetyl-L-Glu
L-Gln
C00169
L-Citrulline
L-Asp
N- (L-arginino) succinate
N-Acetyl ornithine
L-Arg
Fumarate
L-Ornithine
C00086
D
PPP
C02076
C00258
S7P
C00631
C00668
C01172
C00198
C01801
C00672
F6P
C01236
D-gluconic acid
2-deoxy-D-ribose 5-phosphate
F1,6BP
G3P
C00620
C00121
6-phospho-D-gluconate
Ribose 5P
C00279
C00119
C00199
C00231
Pyrimidine metabolism
E
C06198
UDP
UTP
Orotidine 5'-phosphate
S-dihydro orotate
L-Gln
C00169
CTP
UMP
C00119
Orotate
N-Carbamoyl-L-Asp
CDP
dCDP
CMP
dCTP
Cytidine
C00239
C00363
Uridine
Deoxy cytidine
dUMP
C00459
Deoxy uridine
5,6-dihydro uracil
C01346
dTMP
Uracil
3-Ureido propionate
C00460
Thymidine
Beta-Alanine
C00672
Thymine
C21028
C21029
C01205
F
Ala/Asp/Glu metabolism
N-acetyl-L-aspartate
L-Alanine
P5C
Citrate
L-Asp
C02362
D-Asp
N-Acetylaspartyl glutamate
Pyruvate
C00940
L-Glu
C20775
L-Asn
N- (L-arginino) succinate
N-Carbamoyl-L-Asp
C00036
C20776
2-oxoglutarate
4-amino butanoate
L-Gln
Fumarate
C00232
C00014
C00352
C03090
Succinate
C00169

Figure S10

Figure S1) Hsp90s control mitochondrial structure and function. Related to Figure 1.

- **A)** Enlarged images of WT, Hsp90α KO, and Hsp90β KO HEK293 cells incubated with MitoTracker Green (50 nM, 20 m) presented in **Figure 1A**. Scale bar is 10 μm.
- **B)** Additional images of WT, Hsp90α KO, and Hsp90β KO HEK293 cells incubated with MitoTracker Green (50 nM, 20 m) followed by Hoecsht 33258 (10 μg/ml, 10 m). Scale bar is 25 μm.
- **C)** WT, Hsp90α KO, and Hsp90β KO HEK293 cells were incubated with TMRM (50 nM, 20 m). Scale bar is 25 μm.
- **D)** Total number of mitochondria/cell in WT, Hsp90α KO, and Hsp90β KO HEK293 cells based on transmission electron microscopy images presented in **Figure 1B**.
- **E)** Average cristae score per cell based on **Figure 1C**.
- **F)** Enlarged images of representative mitochondria from WT, Hsp90α KO, and Hsp90β KO HEK293 cells.
- **G)** Blue native PAGE (BN-PAGE) of mitochondria isolated from WT, Hsp90α KO, and Hsp90β KO HEK293 cells. Notable ETC complexes have been indicated by labels.
- **H)** Whole cell lysates from WT, Hsp90α KO, and Hsp90β KO HEK293 cells immunoblotted with the indicated antibodies.
- **I)** Densitometric quantification of immunoblots presented in **Figure 1F**. Data are presented as mean ± s.d. *p<0.05, **p<0.005, ***p<0.0005, ****p<.0001 using Student's *t*-test.

Figure S2) Loss of Hsp90α or Hsp90β differentially impacts mitochondrial metabolism. Related to Figure 2.

- **A)** Fold change OCR 4500/1000 mg/L glucose. Calculated from average basal OCR measurements presented in **Figure 2A, 2D**.
- **B)** Proliferation rate of WT, Hsp90α KO, and Hsp90β KO HEK293 cells grown in DMEM containing either 4500 or 1000 mg/L glucose measured by WST Proliferation Assay.

C-H) Fuel Flex assay OCR measurements over time. Measurements used to calculate **Figure 2F-H**.

Figure S3) Metabolic pathway alterations associated with Hsp90 loss. Related to Figure 3.

- **A)** Immunoblot of protein fractions of WT, Hsp90α KO, and Hsp90β KO HEK293 cell samples submitted for metabolomic analysis presented in **Figure 3A-C**.
- **B)** Schematic of interplay between metabolic pathways discussed in this study. Created with Biorender.com
- **C)** Color code of metabolite abundance graphs presented in **Figure S3D-F**. Red: Hsp90α KO (left), Green: Hsp90β KO (center), Blue: Hsp90 WT (right).
- **D)** Impact of Hsp90α or Hsp90β KO on pentose phosphate pathway (PPP),
- **E)** Ala/Asp/Glu metabolism, or
- **F)** TCA cycle flux in HEK293 cells. Color intensity gradient (tan: no significant difference observed, yellow: small difference, orange: intermediate difference, red: large difference) corresponds to weighted impact of metabolite abundance on pathway significance. Created using MetaboAnalyst 6.0.

Figure S4) Hsp90 isoforms regulate diverse aspects of the cellular metabolic network. Related to Figure 3.

- **A)** NAD^+ abundance in metabolite extracts from WT, Hsp90α KO, and Hsp90β KO HEK293 cells, extracted from **Figure 3B**.
- **B)** Densitometric quantification of immunoblots presented in **Figure 3E**. Data are presented as mean ± s.d. *$p<0.05$, **$p<0.005$, ***$p<0.0005$, ****$p<.0001$ using Student's *t*-test.

Figure S5) The mitochondrial metaboproteome is controlled by Hsp90. Related to Figure 4.

- **A)** Immunoblot of cytosolic (C) and mitochondrial (M) fractions of WT and TRAP1 KO HEK293 cells. GAPDH represents a cytosolic control protein, and GRP94 represents a control for

ER contamination. These mitochondria were used for the proteomics study in **Figure 4A-C**.

B) KEGG term enrichment analysis of “mitochondrial protein-containing complex” interaction cluster from **Figure 4A**.

C) Fatty acid oxidation-dependent OCR in WT, Hsp90α KO, and Hsp90β KO HEK293 cells grown in DMEM containing 4500 mg/L glucose. ET - etomoxir

Figure S6) Chaperone compensation supports mitoproteostasis. Related to Figure 5.

A) Interaction network of proteins whose interaction with Hsp90α is upregulated in TRAP1 KO HEK293 cells. Increased color intensity corresponds to degree of interaction upregulation.

B) KEGG enrichment analysis of proteins whose interaction with Hsp90α is upregulated in TRAP1 KO HEK293 cells.

C) Interaction network of proteins whose interaction with Hsp90β is upregulated in TRAP1 KO HEK293 cells. Increased color intensity corresponds to degree of interaction upregulation.

D) KEGG enrichment analysis of proteins whose interaction with Hsp90β is upregulated in TRAP1 KO HEK293 cells.

E) Percent compensation for Hsp90s in TRAP1 KO cells.

F) Interaction network of proteins exhibiting *de novo* Hsp90α interaction in TRAP1 KO cells.

G) KEGG enrichment analysis of proteins exhibiting *de novo* Hsp90α interaction in TRAP1 KO cells.

H) BrdU incorporation assay of WT and TRAP1 KO HEK293 cells grown in DMEM containing 4500 mg/L glucose for 24 h and incubated with 10 μM BrdU for an additional 2 h.

Figure S7) TRAP1 provides partial compensation for Hsp90 isoform loss. Related to Figure 5.

- **A)** Percent chaperone compensation by TRAP1 in Hsp90 KO cells.
- **B)** KEGG enrichment analysis of proteins whose interaction with TRAP1 is upregulated in Hsp90α KO HEK293 cells.
- **C)** KEGG enrichment analysis of proteins whose interaction with TRAP1 is upregulated only in Hsp90α KO HEK293 cells.
- **D)** KEGG enrichment analysis of proteins whose interaction with TRAP1 is upregulated in Hsp90β KO HEK293 cells.
- **E)** KEGG enrichment analysis of proteins whose interaction with TRAP1 is downregulated only in Hsp90α KO HEK293 cells.
- **F)** KEGG enrichment analysis of proteins whose interaction with TRAP1 is downregulated only in Hsp90β KO HEK293 cells. Figures S7A-G are derived from the same experimental dataset and immunoprecipitations were carried out from mitochondrial lysates presented in Figure 1F. n=3 biological replicates per condition.

Figure S8) Metabolic protein assessment in Hsp90 isoform KO cells. Related to Figure 5.

- **A)** Protein levels of a selection of Hsp90- or TRAP1- interacting proteins immunoblotted in whole cell lysates from individual Hsp90 isoform KO cell lines.
- **B)** Levels of a selection of Hsp90- or TRAP1- interacting proteins by immunoblotted in cytosolic (C) or mitochondrial (M) fractions of individual Hsp90 isoform KO cell lines.
- **C)** Relative number of upregulated interactors in chaperone KO cell lines.
- **D)** Chaperone compensation score of Hsp90 isoforms.

Figure S9) Metabolomic evaluation following attenuation of multiple Hsp90 isoforms. Related to Figure 6.

- **A)** Heat map of individual metabolite abundance across each replicate of metabolite extracts from WT or TRAP1 KO HEK293 cells in the presence or absence of G-TPP.

B) UDP-GlcNAc abundance in metabolite extracts from WT or TRAP1 KO HEK293 cells, extracted from **Figure S9A**.

C) Direct comparison of pathway impact of metabolites isolated from WT HEK293 cells in the presence or absence of G-TPP.

D) Direct comparison of pathway impact of metabolites isolated from TRAP1 KO HEK293 cells in the presence or absence of G-TPP.

Figure S10) Metabolic pathway alterations associated with attenuation of multiple Hsp90 isoforms. Related to Figure 6.

A) Color code of metabolite abundance graphs in **Figure 6C**. Red: TRAP1 KO (left), Green: TRAP1 KO + G-TPP (center-left), Blue: TRAP1 WT (center-right), Light blue: TRAP1 WT+G-TPP (right).

B) Impact of TRAP1 and G-TPP treatment on pentose phosphate pathway (PPP),

C) TCA cycle,

D) Pyrimidine metabolism,

E) Arg biosynthesis,

F) Or Ala/Asp/Glu metabolism TCA cycle flux in HEK293 cells. Color intensity gradient (tan: no significant difference observed, yellow: small difference, orange: intermediate difference, red: large difference) corresponds to weighted impact of metabolite abundance on pathway significance. Created using MetaboAnalyst 6.0.
